# Prediction of medication overuse in patients with migraine using cox regression and machine learning: a real-world cohort

**DOI:** 10.1186/s10194-026-02269-3

**Published:** 2026-01-24

**Authors:** Teerapong Aramruang, Pawin Numthavaj, Panu Looareesuwan, Thunyarat Anothaisintawee, Patratorn Kunakorntham, Oraluck Pattanaprateep, Charungthai Dejthevaporn, Ammarin Thakkinstian

**Affiliations:** 1 Department of Clinical Epidemiology and Biostatistics, Faculty of Medicine, Ramathibodi Hospital, Mahidol University, 270 RAMA VI Road, Rachathevi, Bangkok, 10400 Thailand; 2 Department of Pharmacy, Faculty of Pharmacy, Mahidol University, Bangkok, Thailand; 3 Department of Information Technology, Faculty of Medicine, Ramathibodi Hospital, Mahidol University, Bangkok, Thailand; 4 Department of Medicine, Faculty of Medicine, Ramathibodi Hospital, Mahidol University, Bangkok, Thailand

**Keywords:** Migraine, Prediction model, Cox regression, Machine learning, Medication overuse, Medication overuse headache, Real-world data, Electronic health records, Survival analysis

## Abstract

**Background:**

Medication overuse (MO) is a critical issue for patients with migraine, contributing to chronification and medication overuse headache (MOH). Predicting those at risk is essential for effective management. This study aims to develop and compare time-to-event prediction models for MO/MOH among patients with migraine, using a cohort from electronic health records (EHRs).

**Methods:**

A prevalent new-user design of real-world data cohort of patients with migraine conducted at Ramathibodi Hospital, Thailand, from January 2010 to December 2023. The cohort was constructed using EHR data and incorporated common predictors related to the patient, physician, and treatment. Three time-to-event models were developed: Cox proportional hazards (CPH), random survival forests (RSF), and extreme gradient boosting (XGBoost). Model performance was evaluated on a hold-out testing dataset using discrimination and calibration. Variable importance in the machine learning models was assessed using Shapley Additive Explanations.

**Results:**

The study included 13,082 patients with migraine, with 3,456 identified as experiencing MO/MOH, indicating an incidence rate [95% confidence interval (CI)] of 56.31 (54.44–58.21) per 1,000 patient-years. On the testing dataset, the RSF model achieved a concordance index (C-index) of 0.645 (95% CI: 0.643–0.647), slightly outperforming the CPH model’s C-index of 0.635 (95% CI: 0.634–0.636). Additionally, the RSF model recorded the lowest integrated brier score (IBS) of 0.193 (95% CI: 0.192–0.194), compared to 0.195 (95% CI: 0.194–0.196) for the CPH model. The XGBoost model demonstrated lower performance, with a C-index of 0.611 (95% CI: 0.609–0.613) and an IBS of 0.197 (95% CI: 0.195–0.199). Across all models, clinic type, physician position, and history of MO/MOH were significant predictors.

**Conclusions:**

Using a real-world, EHR-derived cohort, we developed time-to-event prediction models incorporating multi-domain predictors to predict MO/MOH in patients with migraine. Although the models demonstrated only modest discrimination, their performance highlights the potential of CPH and machine learning algorithms in this context. External validation and the incorporation of additional clinical predictors, particularly those embedded in unstructured data, are needed.

**Supplementary Information:**

The online version contains supplementary material available at 10.1186/s10194-026-02269-3.

## Background

Migraine is a complex neurological disorder of ten complicated by medication overuse (MO) [1] affecting approximately 15.4% [2]. MO is defined as the regular overuse of one or more acute medications for three months or longer [3], and is a major contributor to chronification [4] and medication overuse headache (MOH) [5], with both terms often used interchangeably. Identifying at-risk patients is essential for guiding preventive treatments and reducing MO/MOH.

A recent systematic review identified six studies presenting nine models to predict MO/MOH in patients with migraine [6]. Three studies developed new prediction models [7–9], while the remaining validated existing scores [10–12]. Although these models demonstrated potential, they relied on a binary classification (non-MO/MOH vs. MO/MOH) without accounting time-to-event data [13]. This limits interpretability, flexibility, and may introduce bias in survival analysis [14]. In contrast, time-to-event models offer dynamic risk estimates over time, thereby enhancing improved tools for monitoring and managing patients with migraine [13].

Moreover, prior studies have faced limitations due to small sample sizes, as indicated by low events per variable (EPV) [6]. According to the Prediction Model Risk of Bias Assessment Tool (PROBAST), a minimum EPV of 10 is recommended, ideally 20–30 events or more per predictor during model development and 100 for external validation [15]. However, only one study met these criteria during score validation [11]. Insufficient sample sizes can lead to model overfitting, selection of unimportant predictors, and overly optimistic performances [16]. Additionally, most studies focused on specific migraine types [9–12] or were restricted to particular clinical settings [7–9, 11, 12], resulting in homogeneous patients that may limit generalizability. Leveraging real-world data (RWD) is essential to overcome these limitations and enhance generalizability.

Electronic health records (EHRs) are valuable sources of RWD, offering rich longitudinal clinical data collected during routine healthcare practice [17]. However, previous studies have underutilized this potential, one study used EHRs only for outcome validation rather than fully leveraging its advantages [8]. As EHRs are primarily designed for routine healthcare management rather than research, clinical data can be dispersed and fragmented across datasets [18]. Therefore, rigorous data processing and integration are essential to effectively harness EHR data for research purposes [19].

To the best of our knowledge, no studies have developed prediction models for MO/MOH in patients with migraine utilizing time-to-event data derived from EHRs. While the Cox proportional hazards (CPH) model remains the standard method for time-to-event analysis due to its familiarity and computational efficiency, it has specific assumptions and limitations [20]. Recent advancements in machine learning (ML) have shown that ML-based models can perform comparably to CPH, particularly by accounting for interaction effects and nonlinear relationships [21, 22]. Among various ML models for survival analysis, random survival forests (RSF) and extreme gradient boosting (XGBoost) have shown good performance. Therefore, this study aims to develop and compare time-to-event prediction models for MO/MOH in patients with migraine using CPH, RSF, and XGBoost models on a large EHR-derived cohort.

## Methods

This study adapted methodological framework from a previous study [19] to suit the specific characteristics of our data. This study also adhered to the Transparent Reporting of a Multivariable Prediction Model for Individual Prognosis or Diagnosis (TRIPOD) [23] and the Guidelines for Developing and Reporting Machine Learning Predictive Models in Biomedical Research [24]. Ethical approval was obtained prior to its initiation (COA No. MURA2023/750).

### Study design and participants

The study was a prevalent new-user design of RWD cohort of patients with migraine treated at the Faculty of Medicine, Ramathibodi Hospital, Mahidol University, Thailand, from January 1, 2010, to December 31, 2023. Patients were eligible if they were adults (aged ≥ 18 years), a diagnosis of migraine between January 1, 2010, and August 31, 2023; received treatments for acute migraine and regular visit at out-patient clinics. Patients were excluded if they had been followed up less than two hospital visits.

### Cohort construction

#### Target patients with migraine identification

**Fig. 1:**
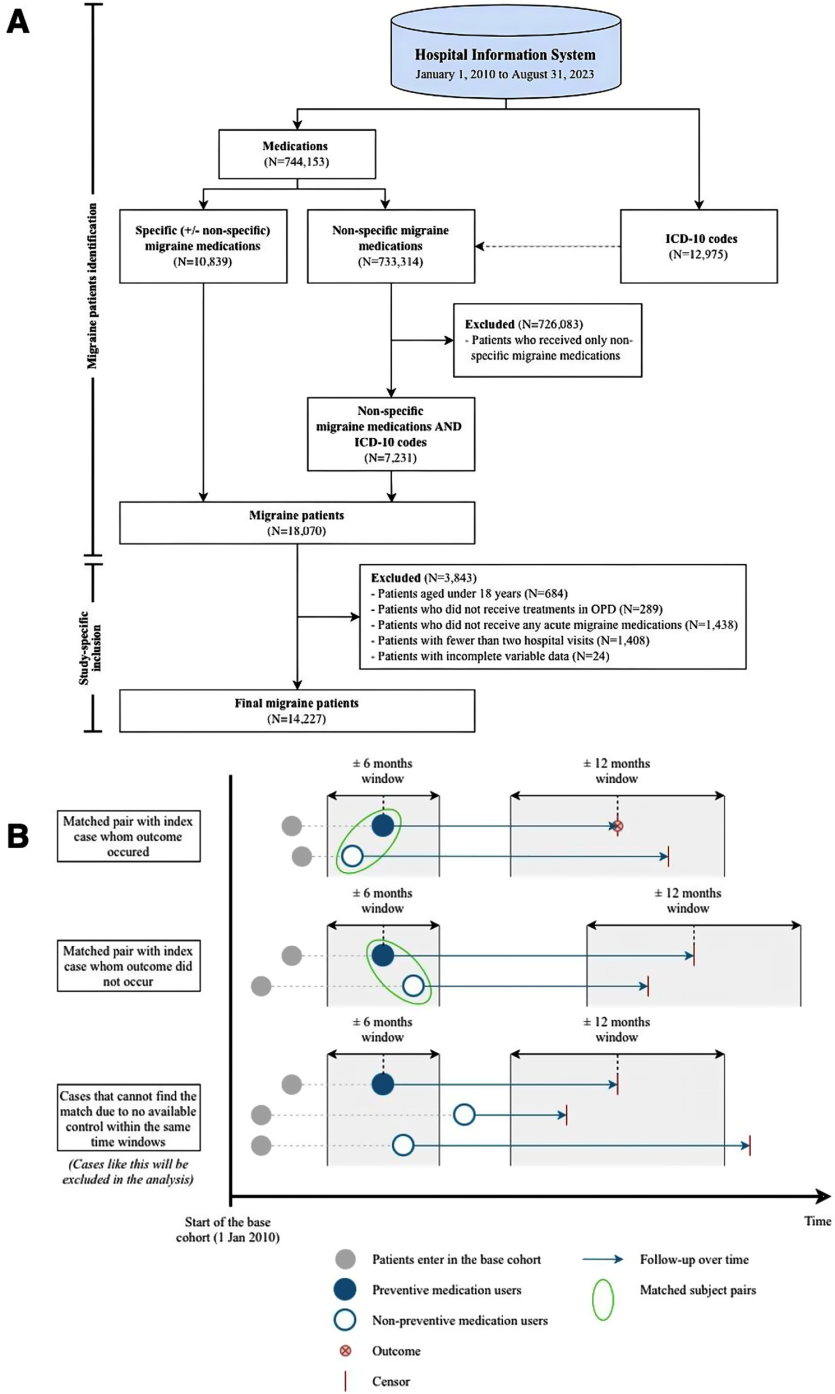
Flow chart for forming the base cohort and the study cohort **(A)** Identification of migraine patients **(B)** Matching process. For example, preventive users, regardless of outcomes or censoring, can be matched with non-preventive users in the first and second pairs since their index dates and follow-up periods fall within designated time windows. In contrast, in the last pair, preventive users cannot be matched with any non-preventive users because their index dates or follow-up periods do not align with the specified time windows, resulting in their exclusion from the analysis

The base cohort was constructed by identifying targeted patients with migraine between January 1, 2010, and August 31, 2023. The steps and criteria for identifying these patients are illustrated in Fig. 1A, and the medications involved in this identification are detailed in Table S1.

The study cohort was constructed using a prevalent new-user design [25, 26] as follows: first, patients who were initially prescribed preventive medications during the study period were identified from the base cohort, then for each preventive medication user, an inactive-control (i.e., patient who did not receive preventive medication) was identified and selected from the same base cohort using time-matched exposure set. Controlled patients were required to be under observation and at risk (i.e., without prior preventive medication) at the time their matched patient initiated preventive medication. Matching was performed chronologically on the preventive-initiation time window within ± 6 months, and the follow-up time (within ± 12 months) with a ratio of 1 case per 1 control, as illustrated in Fig. 1B. Preventive medication users who cannot be matched were excluded from the analysis.

The index date was uniformly defined for both groups as the date of the first preventive medication prescription for preventive users, or the corresponding matched clinic visit date for non-preventive users. This single, harmonized index date was used for both exposure assignment and prediction modelling. Follow-up time was calculated from the index date until the occurrence of an outcome, death, loss to follow-up, or the end of the study period, whichever occurred first. MO/MOH events occurring before the index date were treated as prior history and were not counted as outcomes.

#### Data sources and variables

Data were obtained from three sources: EHRs, human capital, and Clinical Epidemiology and Biostatistics (CEB) data warehouses [27], with detailed descriptions of each source provided in Table S2. Diagnoses and symptoms were coded using the International Classification of Diseases, 10th Revision (ICD-10), while procedures were coded using the International Classification of Diseases, Ninth Revision, Clinical Modification (ICD-9-CM). Data retrieval spanned from January 1, 2010, to December 31, 2023, ensuring that each patient was followed for a sufficient duration, defined as at least 90 days, to assess the outcome of interest.

We included 25 variables associated with MO/MOH in patients with migraine, as suggested by previous studies [2, 28–30] and a systematic review [6]. These selected variables were mapped to the relevant data sources to create metadata that facilitates data standardization and harmonization, focusing on matching variables to structured EHR data.

Physician data, including physician position, graduation period, and years of experience, were requested from the division of human capital because these data could not be obtained from EHRs. The division of human capital is responsible for managing all aspects of the workforce within the hospital. Although hypertension and obstructive sleep apnea could be extracted from the EHRs, we opted to use validated data from the CEB data warehouses, which contain longitudinal cohorts utilizing RWD for non-communicable diseases and are managed by our institute [27]. Consequently, the variables were organized into three domains: patient, physician, and treatment, with the metadata for all variables included in the study detailed in Table S3.

Preventive treatment referred to the initiation of migraine preventive medications prescribed to reduce the frequency and severity of migraine attacks and to prevent MO/MOH. Preventive treatments encompassed the time to initiation of preventive migraine medications and the type of preventive treatments: non-anti-seizure medications (non-ASMs), ASMs, and calcitonin gene-related peptide monoclonal antibodies or combinations of non-ASMs and ASMs.

#### Base cohort establishment

**Fig. 2:**
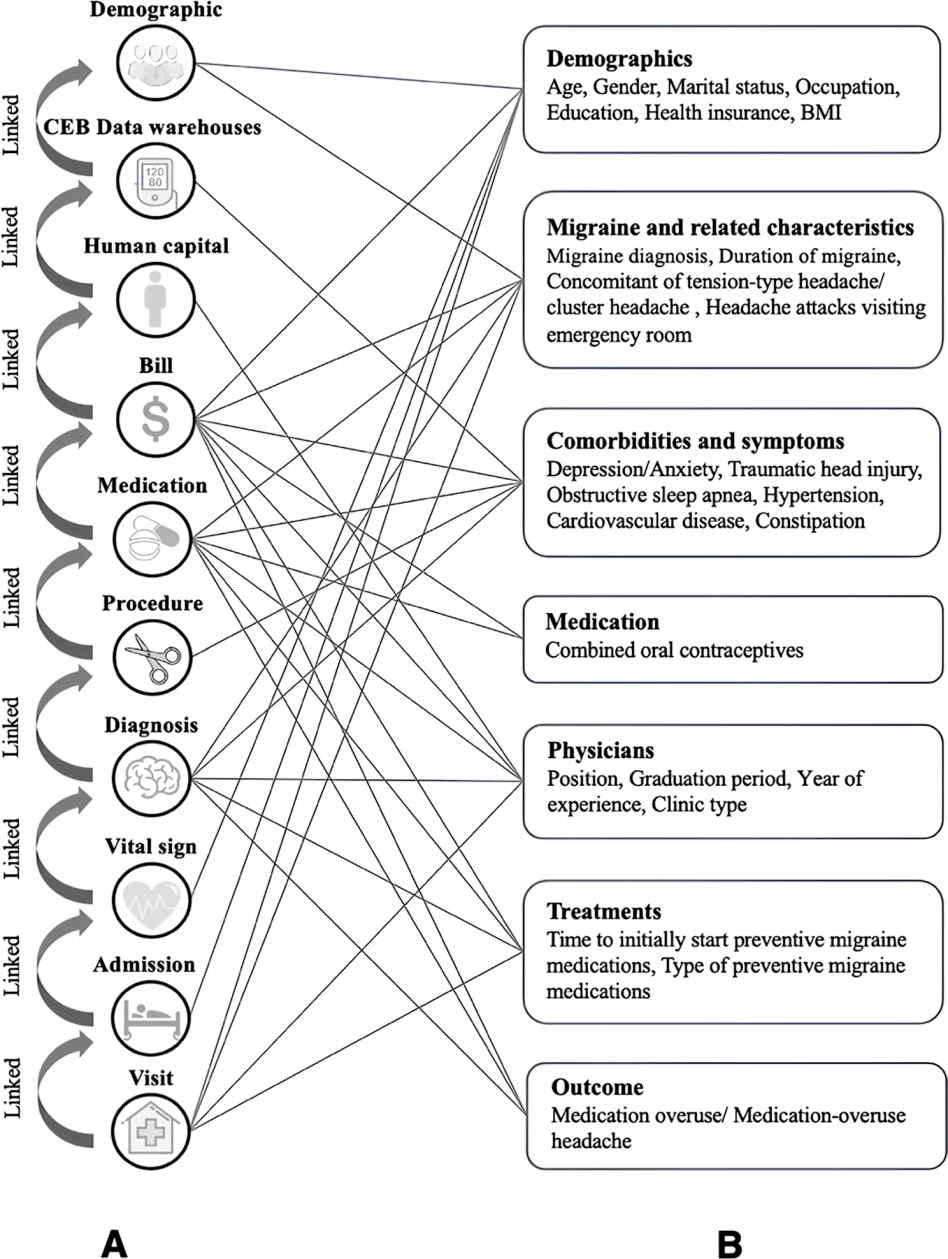
Data sources for establishing the cohort **(A)** Relevant datasets and linking methods employed in the construction of the cohort **(B)** The relationships between the variables and the datasets utilized for variable identification

The base cohort was constructed from patients who initiated acute migraine medications, with cohort entry defined as the date of the first prescription of an acute migraine medication after the diagnosis of migraine (see Figure S1). The data structure retrieved from the EHRs is described in Table S4. Each data domain (demographic, visit, admission, vital sign, diagnosis, procedure, medication, and billing) was processed into a single observation per patient per visit date. These domain-level records were then linked using hashed hospital numbers and visit date to form the base cohort. Physician identifiers embedded within the diagnosis, procedure, medication, and billing data allowed linkage to the human capital dataset to retrieve physician characteristics, as illustrated in Fig. 2A. Following linkage, variable curation and harmonization were conducted based on metadata, and the variables along with the corresponding datasets used for variable identification are shown in Fig. 2B. These included: reconciling variables recorded in more than one domain (e.g., health insurance documented in both demographic and billing data); for which the most frequently observed value (mode) was used when discrepancies occurred; standardizing all date variables to the YYYYMMDD format; grouping medications into standardized drug classes; and ensuring consistent units, categories, and value formats across timepoints and clinicians.

#### Outcomes ascertainment

MO/MOH was defined as the regular overuse of one or more medications for the acute migraine headaches at least three months. Treatments include specific medications (i.e., ergots, triptans, or opioids used for ≥ 10 days per month) and non-specific medications (i.e., non-steroidal anti-inflammatory drugs or paracetamol used for ≥ 15 days per month), following the International Classification of Headache Disorders guideline [3]. The complete list of medications used to identify MO/MOH is provided in Table S5.

MO/MOH was identified by ICD-10 code of G44.4 or prescribing acute medications for more than 90 days. Duration of medication use was calculated by dividing quantity of prescribed medications with a daily dosage. In cases where dosage information was unavailable, we applied the defined daily dose for specific medications, as illustrated in Figure S2.

**Fig. 3:**
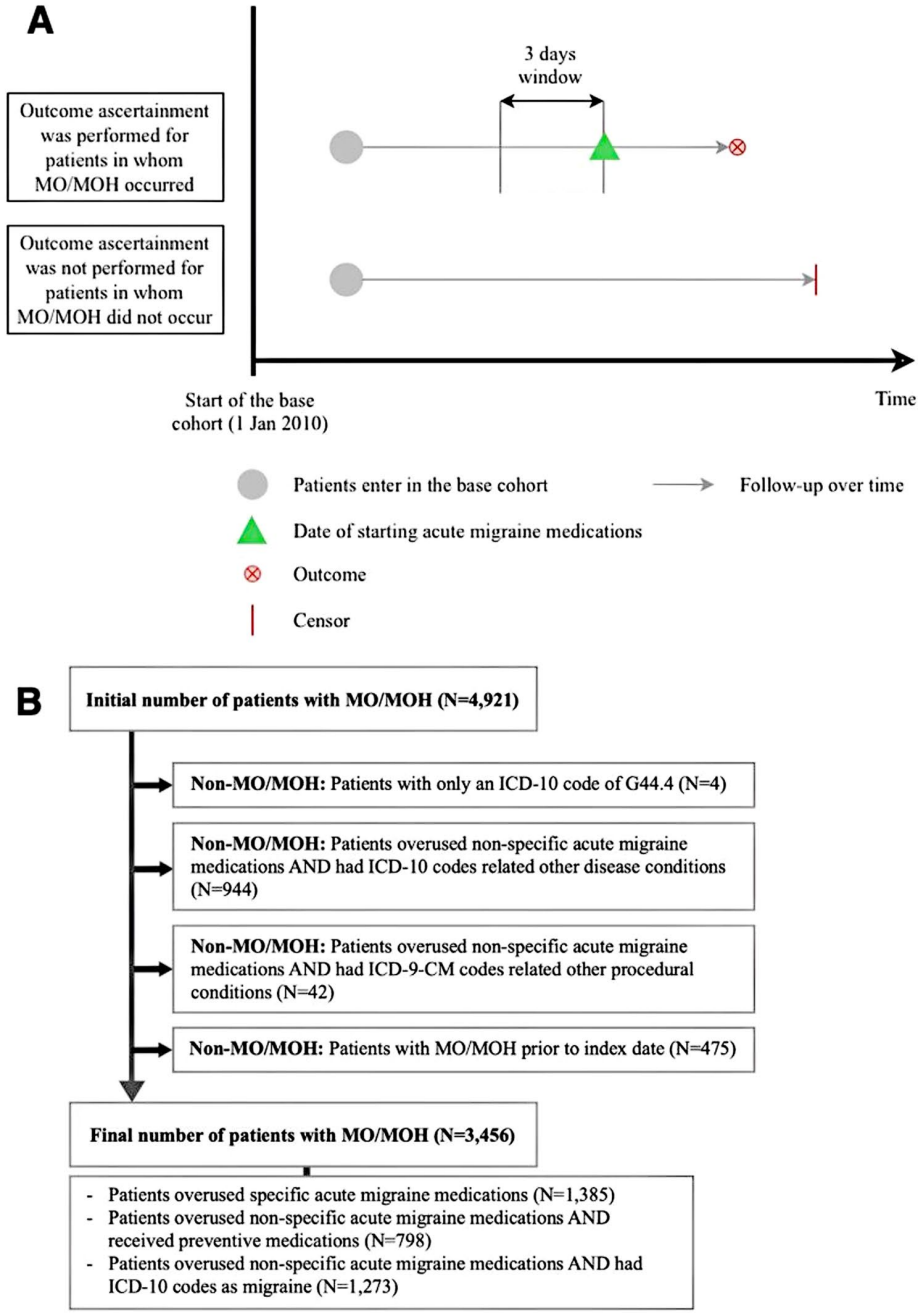
Outcome identification and ascertainment **(A)** Identification period for conducting MO/MOH ascertainment, involving identification of the first day of acute medication prescription causing MO/MOH and tracing back three days **(B)** Steps involved in MO/MOH ascertainment and the corresponding results

To ascertain MO/MOH, acute medication prescriptions were confirmed to be specifically for migraine management based on prescriptions issued within a three-day window. During this period, all relevant ICD-10 and ICD-9-CM codes were extracted and reviewed by a physician to exclude prescriptions related to unrelated indications (see Fig. 3A). The criteria for ascertaining MO/MOH are detailed as follows (see Fig. 3B**)**: (1) Overuse specific acute migraine medications (2) Overused non-specific acute migraine medications in combination with ICD-10 codes diagnosing migraine, with/without preventive medications. Patients were classified as non-MO/MOH if they any of the following: (1) Had only an ICD-10 code of G44.4 without any acute medication use (2) Overused only non-specific acute medications but had ICD-10 or ICD-9-CM codes indicating other pain-related conditions (e.g., osteoarthritis, rheumatoid arthritis, surgical procedures) on the day at prescribing acute medication (3) Had MO/MOH diagnosis before index date in patients received preventive medication. Additionally, for patients with frequent episodes of MO/MOH, manual verification was performed through consultations with a physician to ensure the accuracy of the MO/MOH diagnosis. It is important to note that patients with a MO/MOH prior to the index assessment were not excluded but were classified as non-MO/MOH; their previous history of MO/MOH was used as a predictor in the prediction models.

### Data analysis

To address missing data, we employed two primary approaches: forward and backward carry-over methods and data aggregation over specific time intervals (i.e., 90 days). We subsequently assessed the missing rates for each variable, as describe in Table S6. All variables were complete, except occupation (0.1%), education (0.1%), and body mass index (BMI, 4.6%). Comparing patient characteristics between individuals with complete data (*n* = 14,227) and those with missing data (*n* = 24) indicating no meaningful differences across key demographic or clinical variables; therefore, a complete case analysis was conducted [31, 32]. Variable transformation was then performed, with details available in Table S7.

Baseline variables and outcomes were described using mean and standard deviation or median and interquartile range for continuous variables, and frequency and percentage for categorical variables.

The dataset was randomly split into training and testing datasets with a ratio of 80% and 20%. Three time-to-event models were applied: the CPH model [20], RSF [33], and XGBoost [34]. RSF utilizes a bagging strategy, while XGBoost employs boosting.

Variable selection was performed as follows: A univariate CPH models were fitted in the training dataset to screen candidate predictors. Predictors with a p-value < 0.10 were then considered in the multivariate CPH models using forward selection. Predictors with a p-value < 0.05 were retained in the final multivariate CPH model (called the full model). The proportional hazard (PH) assumption was tested by examining Schoenfeld residuals.

For the ML models, only predictors with a univariate p-value < 0.10 were considered. Model hyperparameters were optimized through stratified 5-fold cross-validation in the training dataset. Discriminative performance, evaluated by a customized concordance index (C-index), was used as the optimization metric.

To enhance reproducibility and generalizability, the reduced models were developed by excluding occupation, health insurance status, physician graduation period, and time to the initiation of preventive migraine medications. Hyperparameter details for both the RSF and XGBoost models (full and reduced models) are provided in Table S8. The performance of the tuned models was then assessed on the testing dataset.

Model performance was evaluated using both discriminative and calibration ability. Bootstrapping was performed by sampling subjects with replacement. The discriminative ability was evaluated by the C-index [35], while the calibration ability was assessed through the integrated brier score (IBS), which is the integral of the brier score across all available time points [36].

The variable importance evaluates the relative contribution of each variable to the predictive power of time-to-event models. In the CPH model, a likelihood ratio test was implemented to assess variable importance [37], and each variable’s contribution is interpreted through hazard ratios (HR) and corresponded variance. For time-to-event ML models, we utilized SHapley Additive exPlanations (SHAP) [38].

Data curation was performed using Python (version 3.8) within the Anaconda environment. Statistical analyses and model development were conducted using Python (version 3.8) and RStudio (version 2024.04.2 + 764).

For the traditional model, the CPH model was implemented using the coxph function from the survival package in R. For the ML models, the RSF models were developed using the RandomSurvivalForest estimator from the scikit-survival library (Python) and the XGBoost model was implemented using xgb function from the xgboost library in Python. Variable importance for the ML models was evaluated using the shap library (Python), which computes SHAP values to quantify predictor contributions.

## Results

### Patient characteristics and outcomes

As illustrated in Fig. 1A, a total of 18,070 patients with migraine were identified between January 1, 2010, and August 31, 2023. After applying study-specific inclusion, the base cohort consisted of 14,227 patients with migraine. Of these, 4,921 were identified as having MO/MOH, four patients with ICD-10 code of G44.4 but no use of acute medication, 944 and 42 patients overused non-specific medications but ICD-10 and ICD-9-CM codes for other pain-related conditions, and 475 had prior history of MO/MOH, leaving 3,456 patients (24.29%) classified as experiencing MO/MOH. As a result, an incidence rate [95% confidence interval (CI)] was 56.31 (54.44–58.21) per 1,000 patient-years.

**Fig. 4:**
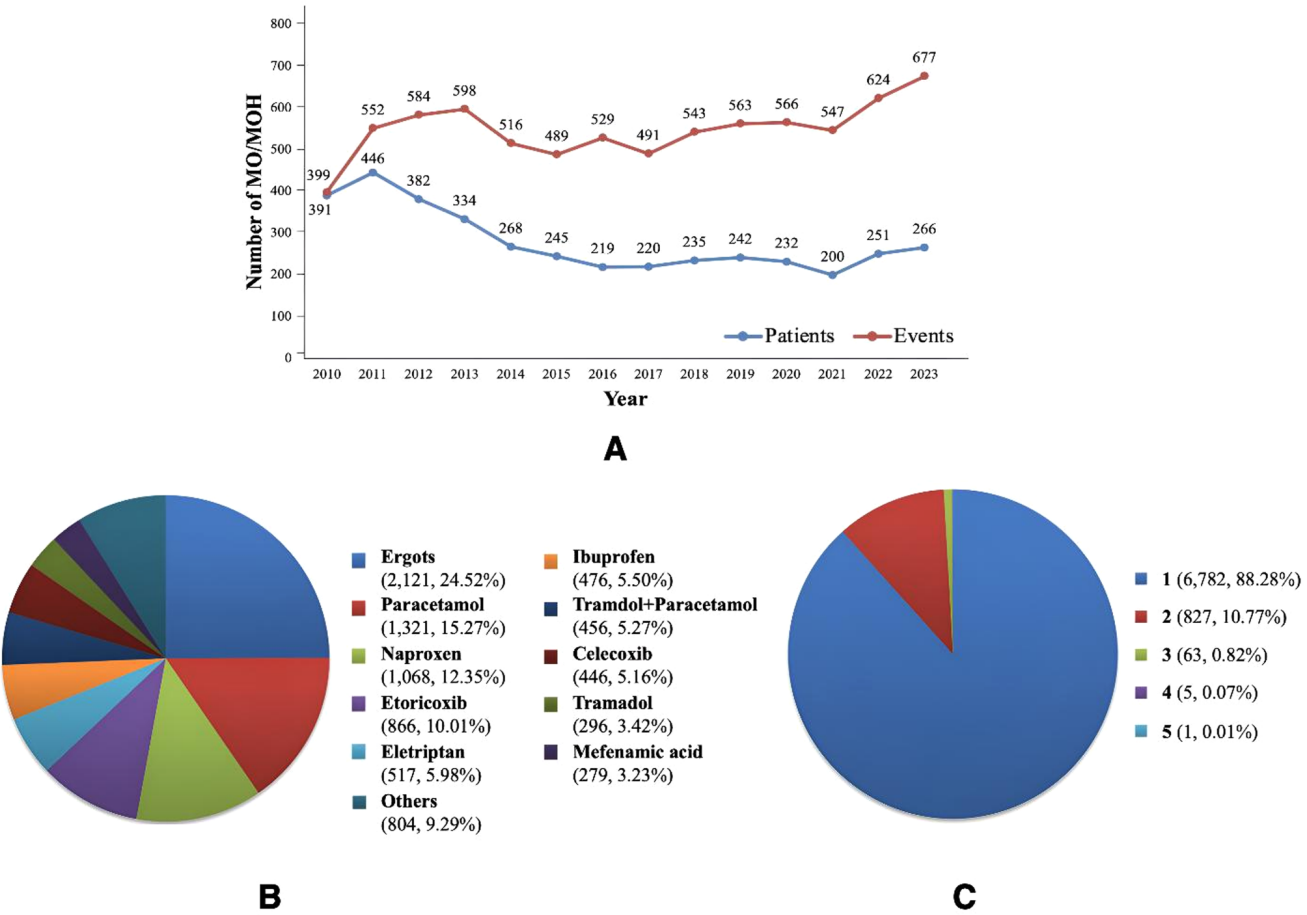
Results of MO/MOH **(A)** Distribution of MO/MOH by patients and events per year **(B)** Proportion of medication types causing MO/MOH events **(C)** Proportion of number of medications causing MO/MOH events

MO/MOH is characterized as a recurrent event, whereby individual patients may experience multiple MO/MOH events, as described in Table S9. The number of MO/MOH episodes ranged from 1 to 15 events, in which most patients experienced only one event (56.58%). The distribution of MO/MOH by patients and events by year is shown in Fig. 4A.

Among the medications contributing to MO/MOH, ergots were the most commonly used (2,121 events, 24.52%), followed by paracetamol (1,321 events, 15.27%) and naproxen (1,068 events, 12.35%), as shown in Fig. 4B. Majority of patients (88.28%) overused a single medication (see Fig. 4C). Only one patient was identified as overusing a combination of five medications, including sumatriptan, diclofenac, mefenamic acid, celecoxib, and tramadol.

Following the prevalent new-user design and matching process, all 6,541 preventive medications were successfully matched with non-preventive patients (6,469 matched within ± 3-months and 72 within ± 6-months of the index date). This yielded a total of 13,082 patients included in the final analysis. The characteristics of patients in the training and testing datasets are summarized in Table 1. Compared with non-MO/MOH patients in the train dataset, those with MO/MOH were slightly older (46.4 vs. 45.1 years) and more commonly female, and a higher civil servant/direct contact insurance (51.9% vs. 42.2%). MO/MOH patients also had higher BMI, shorter migraine duration, and a greater previous MO/MOH (11.1% vs. 4.4%), depression or anxiety (53.3% vs. 42.7%), and hypertension (37.0% vs. 28.1%).

**Table 1:** Characteristics of patients at index assessment in training and testing datasets

Variable	Training dataset (*n* = 10465)			Testing dataset (*n* = 2617)	
	MO/MOH (*n* = 2754)	Non-MO/MOH (*n* = 7711)		MO/MOH (*n* = 702)	Non-MO/MOH (*n* = 1915)
**Patient domain**					
*Demographics*					
Age, year, mean (SD)	46.4 (14.1)	45.1 (15.2)		45.6 (14.5)	45.0 (15.0)
Male, n (%)	367 (13.3)	1184 (15.4)		97 (13.8)	301 (15.7)
Marital status, n (%)					
Married	1461 (53.1)	3638 (47.2)		338 (48.1)	912 (47.6)
Unmarried	1012 (36.7)	3381 (43.8)		294 (41.9)	841 (43.9)
Separated/Divorced/Widowed	281 (10.2)	692 (9.0)		70 (10.0)	162 (8.5)
Occupation, n (%)					
Worker	2095 (76.1)	5671 (73.5)		524 (74.6)	1419 (74.1)
Student	191 (6.9)	813 (10.5)		60 (8.5)	181 (9.5)
Housewife	436 (15.8)	1129 (14.6)		110 (15.7)	295 (15.4)
Retirement	32 (1.2)	98 (1.3)		8 (1.1)	20 (1.0)
Education, n (%)					
Primary school	408 (14.8)	1001 (13.0)		91 (13.0)	224 (11.7)
High school	867 (31.5)	2440 (31.6)		200 (28.5)	615 (32.1)
Bachelor/Higher degree	1479 (53.7)	4270 (55.4)		411 (58.5)	1076 (56.2)
Health insurance, n (%)					
UHC/Social securities	701 (25.5)	2215 (28.7)		184 (26.2)	537 (28.0)
Civil servant/Direct contact	1430 (51.9)	3251 (42.2)		362 (51.6)	804 (42.0)
Self-payment	623 (22.6)	2245 (29.1)		156 (22.2)	574 (30.0)
BMI, kg/m², n (%)					
≤18.4	123 (4.5)	652 (8.5)		30 (4.3)	142 (7.4)
18.5–24.9	1434 (52.1)	4301 (55.8)		380 (54.1)	1075 (56.1)
25.0-29.9	801 (29.1)	1959 (25.4)		201 (28.6)	493 (25.7)
≥30	396 (14.4)	799 (10.4)		91 (13.0)	205 (10.7)
*Migraine and related characteristics*					
Migraine diagnosis, n (%)					
Migraine with aura	32 (1.2)		97 (1.3)	8 (1.1)	33 (1.7)
Migraine without aura	68 (2.5)		206 (2.7)	19 (2.7)	66 (3.4)
Unspecified migraine	2654 (96.4)		7408 (96.1)	675 (96.2)	1816 (94.8)
Duration of migraine, year, n (%)					
<1	2389 (86.7)		6754 (87.6)	612 (87.2)	1695 (88.5)
1–5	315 (11.4)		669 (8.7)	71 (10.1)	164 (8.6)
≥6	50 (1.8)		288 (3.7)	19 (2.7)	56 (2.9)
Concomitant of TTH/CH, n (%)	76 (2.8)		281 (3.6)	14 (2.0)	44 (2.3)
Headache attacks visiting ER, n (%)	101 (3.7)		217 (2.8)	28 (4.0)	69 (3.6)
History of MO/MOH, n (%)	307 (11.1)		337 (4.4)	91 (13.0)	70 (3.7)
*Comorbidities and symptoms*					
Depression/Anxiety, n (%)	1468 (53.3)		3293 (42.7)	380 (54.1)	791 (41.3)
Traumatic head injury, n (%)	10 (0.4)		50 (0.6)	1 (0.1)	12 (0.6)
OSA, n (%)	58 (2.1)		222 (2.9)	11 (1.6)	62 (3.2)
HT, n (%)	1020 (37.0)		2163 (28.1)	243 (34.6)	542 (28.3)
CVD, n (%)	159 (5.8)		421 (5.5)	27 (3.8)	130 (6.8)
Constipation, n (%)	59 (2.1)		149 (1.9)	19 (2.7)	40 (2.1)
*Medication*					
COCs, n (%)	129 (4.7)		476 (6.2)	40 (5.7)	119 (6.2)
**Physician domain**					
Position, n (%)					
Hospitalist/Intern	382 (13.9)		1011 (13.1)	86 (12.3)	276 (14.4)
Resident/Fellow	1277 (46.4)		4025 (52.2)	328 (46.7)	974 (50.9)
Staff	1095 (39.8)		2675 (34.7)	288 (41.0)	665 (34.7)
Graduation period, n (%)					
Before 2006	1223 (44.4)		3068 (39.8)	324 (46.2)	739 (38.6)
After 2006	1531 (55.6)		4643 (60.2)	378 (53.8)	1176 (61.4)
Year of experience, year, median (IQR)	7.0 (1.0–15.0)		7.0 (1.0–16.0)	8.0 (1.0–15.0)	7.0 (1.0–16.0)
Clinic type, n (%)					
Neurology specialist clinic	345 (12.5)		499 (6.5)	75 (10.7)	128 (6.7)
Family medicine clinic	918 (33.3)		3203 (41.5)	236 (33.6)	807 (42.1)
Non-neurology internal medicine specialist clinic	708 (25.7)		1339 (17.4)	188 (26.8)	351 (18.3)
Other specialist clinic	783 (28.4)		2670 (34.6)	203 (28.9)	629 (32.8)
**Treatment domain**					
Preventive migraine medications, n (%)					
No	1105 (40.1)		4108 (53.3)	292 (41.6)	1036 (54.1)
Yes	1649 (59.9)		3603 (46.7)	410 (58.4)	879 (45.9)
Time to initially start preventive migraine medications, month, n (%)					
≤6	1169 (42.4)		2429 (31.5)	293 (41.7)	595 (31.1)
7–12	115 (4.2)		217 (2.8)	27 (3.8)	64 (3.3)
>12	365 (13.3)		957 (12.4)	90 (12.8)	220 (11.5)
Type of preventive migraine medications, n (%)					
Non-ASM	1319 (47.9)		3045 (39.5)	337 (48.0)	741 (38.7)
ASM	198 (7.2)		347 (4.5)	26 (3.7)	91 (4.8)
CGRP mAbs/Combinations	132 (4.8)		211 (2.7)	47 (6.7)	47 (2.5)

Abbreviations: ASM, anti-seizure medication; BMI, body mass index; CGRP, calcitonin gene-related peptide; CH, cluster headache; COCs, combined oral contraceptives; CVD, cardiovascular diseases; ER, emergency room; HT, hypertension; IQR, interquartile range; kg, kilogram; m, meter; mAbs, monoclonal antibodies; MO, medication overuse; MOH, medication overuse headache; n, number; OSA, obstructive sleep apnea; SD, standard deviation; TTH, tension-type headache; UHC, universal health coverage

Regarding healthcare utilization, MO/MOH patients also attended follow-up appointments more often with staff physicians (39.8% vs. 34.7%) and visited neurology (12.5% vs. 6.5%) and non-neurology internal medicine clinics (25.7% vs. 17.4%) more frequently. Additionally, MO/MOH patients had higher rates of receiving preventive medications (59.9% vs. 46.7%), with most receiving non-ASMs (47.9% vs. 39.5%) for prevention.

### Variable importance

**Fig. 5:**
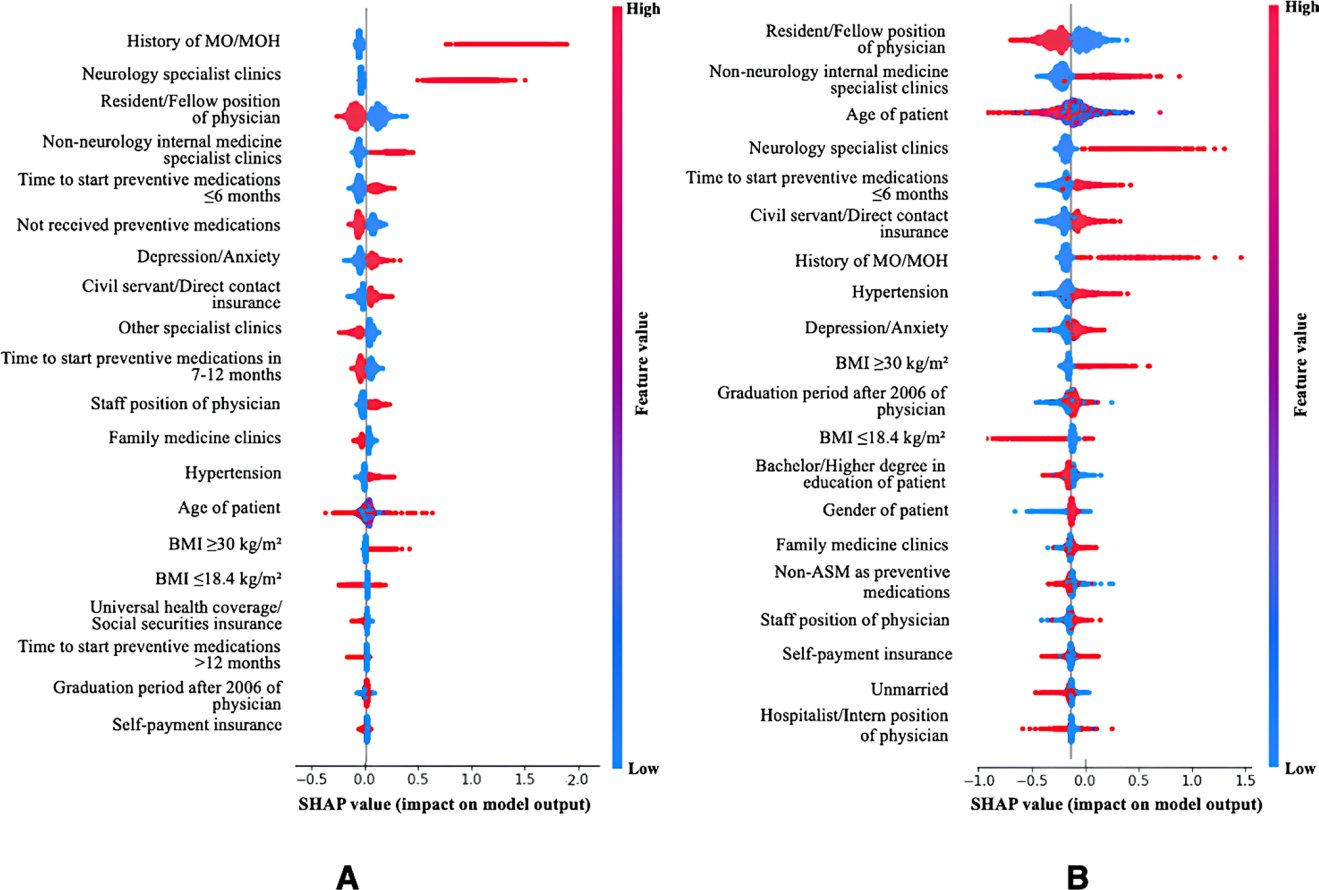
Summary plots of variable importance analysis for **(A)** RSF model and **(B)** XGBoost model (Full model), utilizing SHapley Additive exPlanation (SHAP) for the top 20 most significant variables selected from the training dataset. The X-axis of the graph represents the impact of each variable on the prediction results, while the Y-axis lists the model variables. A higher position on the graph indicates a stronger correlation between the variable and the prediction outcome. Blue colors represent low variable values, whereas pink colors signify high variable values

From the initial 25 variables evaluated in the univariate analysis (see Table S10), 20 variables with p-values < 0.10 were selected for inclusion in the multivariate CPH and ML models. The final full CPH model incorporated 15 significant variables, as detailed in Table 2. The top five predictors of MO/MOH, identified through a likelihood ratio test, were clinic type, history of MO/MOH, physician position, insurance, and BMI (all with p-values < 0.001). For MLs, variable importance was evaluated by SHAP, as shown in Fig. 5. Clinic type, physician position, and time to initially start of preventive migraine medications consistently ranked among the top five most important variables across all models.

**Table 2:** Predictive variables of MO/MOH: A multivariate Cox proportional hazards model

Predictive variables	Full model			Reduced model		
	HR (95% CI)	SE	*P*-value	HR (95% CI)	SE	*P*-value
**Patient domain**						
*Demographics*						
Age, year	0.99 (0.98–0.99)	0.00	< 0.001	0.99 (0.98–0.99)	0.00	0.001
Gender						
Male	1			1		
Female	1.11 (1.01–1.23)	0.05	0.037	1.11 (1.01–1.22)	0.05	0.049
Education						
Primary school	1			1		
High school	0.90 (0.81–1.01)	0.06	0.062	0.92 (0.83–1.03)	0.06	0.143
Bachelor/Higher degree	0.84 (0.75–0.93)	0.05	< 0.001	0.90 (0.82–0.98)	0.05	0.036
Health insurance						
UHC/Social securities	1			NA		
Civil servant/Direct contact	1.33 (1.22–1.46)	0.05	< 0.001	NA	NA	NA
Self-payment	1.00 (0.91–1.11)	0.05	0.968	NA	NA	NA
BMI, kg/m²						
≤18.4	1			1		
18.5–24.9	1.41 (1.20–1.66)	0.08	< 0.001	1.43 (1.21–1.67)	0.08	< 0.001
25.0-29.9	1.53 (1.29–1.81)	0.09	< 0.001	1.56 (1.31–1.84)	0.09	< 0.001
≥30	1.78 (1.49–2.12)	0.09	< 0.001	1.80 (1.50–2.15)	0.09	< 0.001
*Migraine and related characteristics*						
Duration of migraine, year						
<1	1			1		
1–5	1.53 (1.19–1.96)	0.13	< 0.001	1.53 (1.19–1.95)	0.13	< 0.001
≥6	1.72 (1.35–2.16)	0.13	< 0.001	1.71 (1.34–2.16)	0.13	0.001
Concomitant of TTH/CH						
No	1			1		
Yes	0.78 (0.63–0.95)	0.11	0.016	0.79 (0.64–0.96)	0.10	0.020
History of MO/MOH						
No	1			1		
Yes	2.24 (2.01–2.49)	0.05	< 0.001	2.24 (2.01–2.49)	0.06	< 0.001
*Comorbidities and symptoms*						
Depression/Anxiety						
No	1			1		
Yes	1.23 (1.13–1.33)	0.04	< 0.001	1.22 (1.13–1.34)	0.04	< 0.001
Hypertension						
No	1			1		
Yes	1.23 (1.14–1.33)	0.04	< 0.001	1.23 (1.14–1.33)	0.04	< 0.001
OSA						
No	1			1		
Yes (without CPAP)	0.76 (0.60–0.96)	0.13	0.023	0.75 (0.59–0.95)	0.13	0.019
Yes (with CPAP)	0.30 (0.10–0.95)	0.58	0.038	0.31 (0.10–0.95)	0.58	0.037
**Physician domain**						
Position						
Hospitalist/Intern	1			1		
Resident/Fellow	0.85 (0.76–0.95)	0.06	0.004	0.84 (0.76–0.94)	0.06	0.003
Staff	1.26 (1.12–1.42)	0.06	< 0.001	1.21 (1.08–1.35)	0.06	0.002
Graduation period						
Before 2006	1			NA		
After 2006	1.08 (1.01–1.16)	0.04	0.048	NA	NA	NA
Clinic type						
Neurology specialist clinic	1			1		
Family medicine clinic	0.55 (0.49–0.62)	0.06	< 0.001	0.55 (0.49–0.62)	0.06	< 0.001
Non-neurology internal medicine specialist clinic	0.80 (0.71–0.91)	0.06	< 0.001	0.79 (0.70–0.89)	0.06	< 0.001
Other specialist clinic	0.52 (0.46–0.58)	0.06	< 0.001	0.52 (0.46–0.58)	0.06	< 0.001
**Treatment domain**						
Type of preventive migraine medications						
Not received	1			1		
Non-ASM	0.69 (0.54–0.87)	0.13	0.002	0.68 (0.54–0.86)	0.13	0.001
ASM	0.74 (0.56–0.97)	0.15	0.031	0.72 (0.55–0.95)	0.14	0.020
CGRP mAbs/Combinations	0.76 (0.57–0.98)	0.15	0.045	0.74 (0.55–0.96)	0.15	0.035

Abbreviations: ASM, anti-seizure medication; BMI, body mass index; CGRP, calcitonin gene-related peptide; CH, cluster headache; CI, confidence interval; COCs, combined oral contraceptives; CPAP, continuous positive airway pressure; HR, hazard ratio; HT, hypertension; kg, kilogram; m, meter; mAbs, monoclonal antibodies; MO, medication overuse; MOH, medication overuse headache; NA, not applicable; OSA, obstructive sleep apnea; SE, standard error; TTH, tension-type headache; UHC, universal health coverage

Specifically, the full CPH model indicated that patients treated in family medicine, non-neurology internal medicine specialist, and other specialist clinics had decreased MO/MOH risks of 0.55 (95% CI: 0.49–0.62), 0.80 (95% CI: 0.71–0.90), and 0.52 (95% CI: 0.46–0.58), respectively, compared to those in neurology specialist clinics. Patients treated by residents/fellows had about 15% [HR = 0.85 (95% CI: 0.76–0.95)] lower risk than those treated by hospitalists/interns. Conversely, care from staff physicians increased MO/MOH risk about 26% [HR = 1.26 (95% CI: 1.12–1.42)] compared to care from hospitalists/interns. A history of MO/MOH increased the risk by 2.24 times (95% CI: 2.01–2.49), consistent with findings from the RSF model. Insurance and BMI ranked among the top five variables for importance only within the CPH model, while patient age were top ranked exclusively in the XGBoost model. Additional details on variable importance are provided in Figure S3 and Table S11.

In the reduced models, 13 out of 15 variables remained in the final CPH model, indicating similar top four important predictors (clinic type, history of MO/MOH, physician position, BMI) plus hypertension and also HRs, see Table 2 and Table S11. The PH assumption was assessed for all CPH models. Both the full and reduced CPH models demonstrated violations of the PH assumption, see Table S12.

### Model performance

The evaluation results for the C-index and IBS are detailed in Table 3. In the full models, both the CPH and RSF models demonstrated comparable overall performance, although the RSF model showed slightly higher discrimination than the CPH model [C-index: 0.645 (95% CI: 0.643–0.647) vs. 0.635 (95% CI: 0.634–0.636)] on the testing dataset, and a marginally better fit, reflected by a lower IBS value [0.193 (95% CI: 0.192–0.194) vs. 0.195 (95% CI: 0.194–0.196)]. The XGBoost model showed the lowest performance across both metrics, with a C-index of 0.611 (95% CI: 0.609–0.613) and an IBS of 0.197 (95% CI: 0.195–0.199) on the testing dataset.

**Table 3:** Model performance on both training and testing datasets

Model	C-index (95% CI)		IBS (95% CI)	
	Training dataset	Testing dataset	Training dataset	Testing dataset
*Full models*				
CPH	0.649 (0.648–0.650)	0.635 (0.634–0.636)	0.194 (0.193–0.195)	0.195 (0.194–0.196)
RSF	0.665 (0.663–0.667)	0.645 (0.643–0.647)	0.186 (0.185–0.187)	0.193 (0.192–0.194)
XGBoost	0.642 (0.640–0.644)	0.611 (0.609–0.613)	0.187 (0.186–0.188)	0.197 (0.195–0.199)
*Reduced models*				
CPH	0.644 (0.643–0.645)	0.634 (0.633–0.635)	0.193 (0.192–0.194)	0.194 (0.193–0.195)
RSF	0.661 (0.660–0.662)	0.636 (0.635–0.637)	0.187 (0.185–0.189)	0.191 (0.189–0.193)
XGBoost	0.637 (0.635–0.639)	0.598 (0.597-0.600)	0.188 (0.187–0.189)	0.196 (0.195–0.197)

Abbreviations: CI, confidence interval; C-index, concordance index; CPH, Cox proportional hazard; IBS, integrated brier score; RSF, random survival forests; XGBoost, extreme gradient boosting

In the reduced models, similar discriminative performance was observed in both the CPH and RSF models [0.636 (95% CI: 0.635–0.637) vs. 0.634 (95% CI: 0.633–0.635)] on the testing dataset, Table 3. However, the RSF demonstrated slightly better calibration/fit with lower IBS compared to the CPH model [0.191 (95% CI: 0.189–0.193) vs. 0.194 (95% CI: 0.193–0.195)]. The XGBoost model again perform lowest, with a C-index of 0.598 (95% CI: 0.597-0.600) and an IBS of 0.196 (95% CI: 0.195–0.197) on the testing dataset. Although the reduced models exhibited a modestly lower C-index than the full models, they demonstrated lower IBS suggesting better calibration and less overfitting.

Model stability was assessed by comparing performance metrics between the training and testing datasets. For discrimination, the differences in C-index between testing and training datasets were − 0.014 (CPH), -0.020 (RSF), and − 0.031 (XGBoost) in the full models, and − 0.010 (CPH), -0.025 (RSF), and − 0.039 (XGBoost) in the reduced models. These findings indicate that the CPH model exhibited the highest stability, with the smallest performance drop from training to testing.

A similar trend was observed for calibration. The changes in IBS between training and testing datasets were 0.001 (CPH), 0.007 (RSF), and 0.010 (XGBoost) in the full models, and 0.001 (CPH), 0.004 (RSF), and 0.008 (XGBoost) in the reduced models. Again, the CPH model demonstrated the greatest stability, while RSF and XGBoost showed larger deviations.

## Discussion

We developed time-to-event prediction models using both traditional CPH and ML approaches. Overall, our results demonstrated comparable discrimination and calibration performance between CPH and RSF models, while XGBoost model showed the lowest performance. In the full model, RSF model slightly outperformed the CPH model in both discrimination and calibration. However, RSF model exhibited greater performance decline between training and testing datasets compared with the CPH model, indicating reduced stability. In contrast, the CPH model displayed the smallest decreases in both C-index and IBS, indicating that it was the most stable across datasets.

A key methodological consideration is that both the full and reduced CPH models violated the PH assumption, indicating that the effects of several predictors on the outcome were not constant overtime. Such violations challenge the validity of CPH model in this context and may limit its generalizability particularly when applied to external datasets where time-varying effects may differ. In addition, the CPH model assumes linear relationships between covariates and log hazards, which may underestimate/overestimate risks in settings where nonlinear effects or interactions present [20].

Conversely, the RSF and XGBoost models are not constrained by the PH assumption and can flexibly accommodate nonlinear relationships and higher-order interactions without requiring explicit programming [21, 22]. These strengths typically make ML-based survival models advantageous in settings with diverse/complex predictors-outcome relationships or when time-varying effects are anticipated. However, despite the PH assumption violations and the theoretical flexibility of ML models, the performance of RSF and XGBoost in our study was not substantially superior to that of CPH. This suggests that nonlinearities and time-varying effects, although present, may not have exerted a strong influence on predictive accuracy in this dataset. Alternatively, the CPH model’s robustness may reflect the underlying structure of the data, where the bias arising from PH violations had limited impact on predictive accuracy.

Since this study is the first to develop time-to-event prediction models for MO/MOH in patients with migraine, direct comparisons of model performance with previous work are challenging. Previous research comparing CPH and ML models in other clinical domains yielded inconsistent results in their comparisons [39, 40]. Therefore, it is necessary to further investigate and better understand circumstances in which ML methods may offer meaningful advantages over the CPH model. Our findings also highlight the need for future studies to incorporate modelling strategies that explicitly address time-varying covariates (e.g., time-varying CPH) [41] or extending to neural survival models capable of capturing complex temporal dynamics [42].

To address potential temporal inconsistencies and minimize immortal-time bias, we analyzed the study cohort with a prevalent new-user design [25, 26]. Patients who initiated preventive medication were matched 1:1 with inactive comparators who had not yet received preventive therapy at a similar calendar time. Matching was performed chronologically on preventive initiation time within ± 6 months, with comparable follow-up windows (± 12 months), ensuring that both preventive users and non-users were under observation and at risk at cohort entry. The time zero or the uniformed index date of both groups was properly defined. This design choice was motivated by the recognition that immortal time (commonly introduced when exposure status is defined after cohort entry) can lead to biased effect estimates and overestimation of treatment benefits in observational studies. By aligning cohort entry, exposure assignment, and follow-up across groups, the prevalent new-user design strengthens causal interpretability while maintaining real-world clinical relevance [25, 26]. However, it is important to note that this approach results in the exclusion of unmatched patients, leading to a reduction in sample size.

In addition, this study is the first to incorporate multi-faceted predictors including patient, physician, and treatment, contrasting with previous studies that primarily focused on only patient-related variables [6]. Notably, the inclusion of treatment predictors aligns with the TRIPOD recommendations, which emphasize the importance of integrating treatment-related predictors to enhance a model’s validity and applicability [23].

Clinic type and physician position consistently emerged as key predictors across all models; however, these variables are rarely reported in the existing literature. Notably, our findings indicate that patients treated in family medicine, non-neurology internal medicine specialist, and other specialist clinics have a lower risk of MO/MOH compared to those treated in neurology specialist clinics. This may align with previous research showing that patients with migraine consulting neurologists or headache specialists have an odds ratio of 1.93 for inadequate acute treatment response. These patients often require additional doses of medications for headache, increasing the incidence of MOH. Another reason may arise from inadequate responders are more likely to seek specialist care, as these specialists typically handle more severe cases [43] and have significantly lower rates of delayed diagnosis compared to other medical specialists [44]. Additionally, differences in prescription patterns for preventive medications between neurologists and other specialists may further contribute to MO/MOH [45]. In our setting, most patients with migraine initially seek care at family medicine clinics. Those with severe cases are referred to neurology specialist clinics. Typically, referred patients have already experienced MO/MOH, assigning neurologists the responsibility of managing severe cases, potentially involving developed or existing MO/MOH. Physician position is also associated with MO/MOH risk; prior study suggests that specialists (i.e., staff positions) and increased years of experience possess greater knowledge and competency of migraine management compared to general practitioners and residents, which may result in staff positions treating severe cases at higher risk for MO/MOH [46]. Additionally, a history of MO/MOH was linked to a 2.24-fold increase in MO/MOH risk, supporting findings that 42% of patients with MOH experience headache relapses within three years [5]. Notably, XGBoost model identified different critical predictors than CPH model; this discrepancy may reflect the ability of ML models to uncover complex relationships within the data that traditional statistical methods might overlook.

The cohort included all types of patients with migraine from diverse clinical settings, enhancing representation of the real-world population. This was accomplished through the use of RWD sourced from EHRs [19], in accordance with TRIPOD recommendations [23]. Collecting data under real-world conditions improves the representativeness and generalizability of findings, reduces costs and effort, and allows for the capture of large populations and events over extended periods [47]. This study employed a large cohort of 14,227 patients, yielding an EPV of 138.4, consistent with PROBAST recommendations [15], and represents the largest sample size reported in comparison to previous studies [6].

However, EHRs present several inherent challenges [17]. In our study, we encountered minimal missing data, which allowed us to perform a complete case analysis. In contrast, studies with higher missing data rates may employ more robust methods, such as Multivariate Imputation by Chained Equations [48], to maintain sample size. EHRs also pose difficulties with newly approved drugs, where routine data may be limited. For instance, calcitonin gene-related peptide monoclonal antibodies were introduced in the hospital around 2020, resulting in only 22 patients receiving these treatments; this small minority class for specific variables could lead to convergence issues during model development. Furthermore, as patients have been visited over the past decade, advancements in treatment options and update in clinical practice guidelines for management of patients with migraine may have influenced prognosis and, consequently, model stability.

To effectively utilize EHRs, we adapted methodologies from a prior study to develop reliable a cohort [19]. We meticulously implemented processes to ascertain MO/MOH, and our findings revealed that ergots, paracetamol, and naproxen were the most frequently overused medications, consistent with previous studies [49, 50]. However, the selection of acute medications is influenced by various factors. We included variables extracted from EHRs, which are routinely available in clinical practice, unlike previous studies that included variables requiring specialized measurements (e.g., questionnaire-based evaluations, genetic testing) [6]. This approach aligns with PROBAST recommendations that predictors in prediction models should be accessible at the point of application [15]. However, inclusion of certain health system (e.g., health insurance) and context specific predictors may limit generalizability of the full models to settings with different healthcare structures. To address this, we developed reduced models excluding such predictors. These reduced models achieved performance comparable to the full models, supporting their potential utility in broader clinical contexts.

To our knowledge, this study is the first to develop time-to-event models for predicting MO/MOH in patients with migraine using a large cohort derived from EHRs. Our findings aim to address this gap and serve as a reference for future research. Despite using rigorous methodologies to create reliable models, several limitations must be acknowledged. First, the cohort is sourced from EHRs primarily designed for healthcare management, which may result in misidentification of target patients, although hierarchical steps to mitigate misidentification was applied during patient identification. Second, some variables associated with MO/MOH were omitted, as we relied on only structured EHR data but excluding unstructured information. Important variables, such as monthly headache days and physical activity [2, 8, 29], were recorded as narrative text and not directly retrievable. Future studies should leverage large language models to extract and structural information from unstructured clinical data, which may enhance model performance beyond the modest discrimination observed in this study [51]. Third, our analysis utilized EHR data from our setting, making it challenging to determine diagnosis dates for patients transferred from other hospitals, and since our outcome relies on medication data, it may not capture comprehensive treatment information if patients receive care outside our facility. Fourth, outcome identification assumes that the quantity of medications dispensed reflects actual consumption. While indirect adherence assessment methods using EHR data are user-friendly, inexpensive, and non-invasive, they primarily show dispensing rather than ingestion [52]. We base our MO/MOH estimation on this assumption, which may potentially lead to an overestimation of MO/MOH. Lastly, although the cohort is relatively large, it is drawn from a tertiary care hospital in Bangkok, Thailand, which may not represent the broader Thai population. Nonetheless, our findings may be applicable to other similar tertiary care settings across the country and Southeast Asia with comparable insurance structures and access patterns. Genetic factors associated with MO/MOH risk, such as serotonin transporter and methylenetetrahydrofolate reductase polymorphisms [7], may also differ by populations, potentially limiting generalizability to Western contexts. External validation in independent cohorts is needed, and we plan to conduct this in future work.

## Conclusions

This study developed time-to-event prediction models that incorporate multi-domain predictors to predict MO/MOH in patients with migraine, utilizing a real-world cohort derived from EHRs. The models demonstrated modest discrimination, highlighting the potential of CPH and ML approaches in this context. However, further enhancement requires external validation and the integration of additional clinical predictors, particularly those embedded in unstructured data.

## Supplementary Information

Below is the link to the electronic supplementary material.

Supplementary Material 1

## Data Availability

The datasets used and/or analysed during the current study are available from the corresponding author on reasonable request.

## Abbreviations

ASMs: Anti-seizure medications
BMI: Body mass index
CEB: Clinical Epidemiology and Biostatistics
C-index: Concordance index
CI: Confidence interval
CPH: Cox proportional hazards
EHR: Electronic health record
EPV: Events per variable
HR: Hazard ratios
IBS: Integrated Brier score
ICD-10: The International Classification of Diseases, 10th Revision
ICD-9-CM: The International Classification of Diseases, Ninth Revision, Clinical Modification
ML: Machine learning
MO: Medication overuse
MOH: Medication overuse headache
PH: Proportional hazard
PROBAST: The Prediction Model Risk of Bias Assessment Tool
RSF: Random survival forests
RWD: Real-world data
SHAP: SHapley Additive exPlanations
TRIPOD: The Transparent Reporting of a Multivariable Prediction Model for Individual Prognosis: or Diagnosis
XGBoost: Extreme gradient boosting
