# Supplementary material for: Prediction of medication overuse in patients with migraine using cox regression and machine learning: a real-world cohort
Source: J Headache Pain. 2026 Jan 24;27(1):46. doi: 10.1186/s10194-026-02269-3 (PMC12911303; doi:10.1186/s10194-026-02269-3)
Supplement: Supplementary file 1 — Supplementary Material 1 [file 10194_2026_2269_MOESM1_ESM.pdf]

SUPPLEMENTAL MATERIAL

Supplementary Figure Legends

Figure S1 Defining the first index date or the date enter to the base cohort

Figure S2 Steps for identifying MO/MOH through the medication dataset using the carry-forward method

Figure S3 Variable importance of ML models (Reduced model)

Supplementary Table Legends

Table S1 List of medications used in this study

Table S2 Description of data sources for constructing the cohort in the study

Table S3 The metadata for the variables included in the study

Table S4 Structure of the data retrieved from the EHRs

Table S5 Definitions and measurement methods for outcome in the study

Table S6 Missing assessment of variables and characteristics of patients at index assessment for those included in the final analysis and those excluded due to missing variable data

Table S7 Variable type and transformation

Table S8 Lists of hyperparameters for tuning the RSF and XGBoost models

Table S9 Number of patients corresponding to each number of MO/MOH events experienced

Table S10 Univariate CPH model on training dataset

Table S11 Variable importance of CPH model using likelihood ratio test on training dataset

Table S12 CPH assumption tested with Schoenfeld residuals

Figure S1 Defining the first index date or the date enter to the base cohort

The first index date refers to the date on which patients with migraine entered the base cohort, defined as the initial date when a patient began using acute medications following their first diagnosis of migraine.

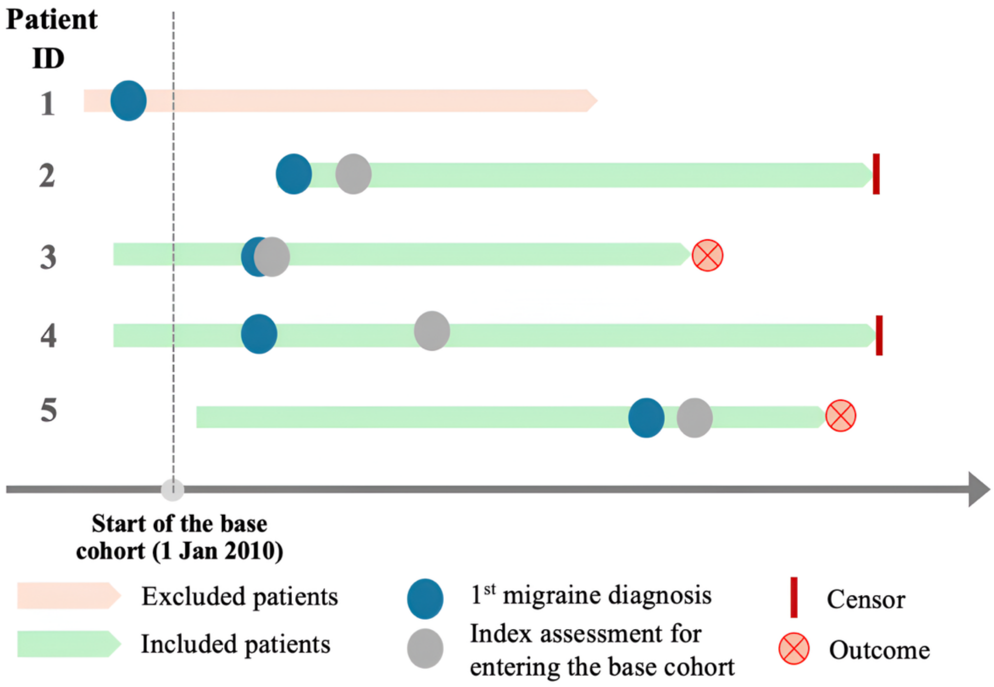

Figure S2 Steps for identifying MO/MOH through the medication dataset using the carry-forward method

Patient ID	Day	Quantity of drug
1	1	100
1	30	
1	60	
1	90	
1	120	
2	1	70
2	30	
2	60	
2	90	

Carry forward

Patient ID	Day	Quantity of drug	Take drug
1	1	140	1
1	30		1
1	60		1
1	90		1
1	120		1
2	1	70	1
2	30		1
2	60		1
2	90		0

Figure S3 Variable importance of ML models (Reduced model)

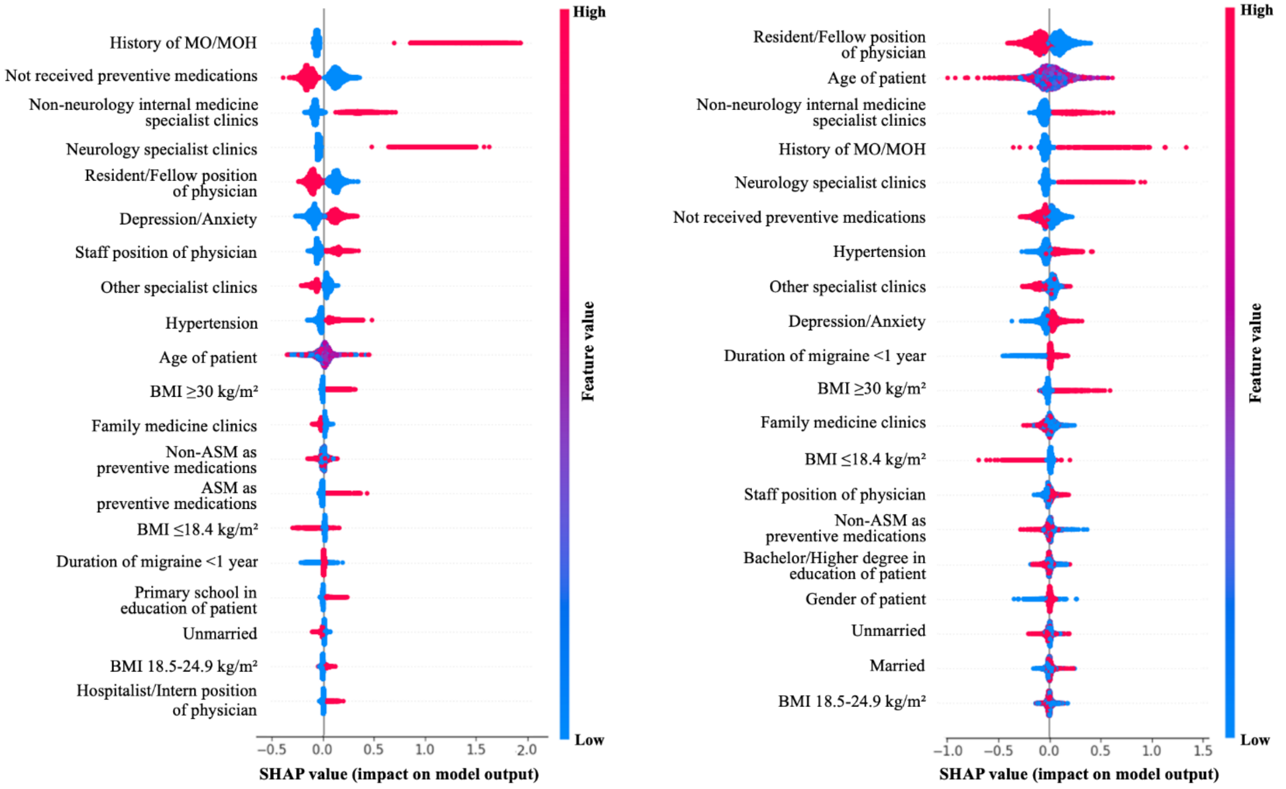

A **B**

Summary plot of variable importance analysis for **(A)** RSF model and **(B)** XGBoost model (Reduced model), utilizing SHAP for the top 20 most significant variables selected from the training dataset. The X-axis of the graph represents the impact of each variable on the prediction results, while the Y-axis lists the model variables. A higher position on the graph indicates a stronger correlation between the variable and the prediction outcome. Blue colors represent low variable values, whereas pink colors signify high variable values.

Table S1 List of medications used in this study

Medications used for identifying target patients with migraine from the EHRs

**Non-specific migraine**
Acute medication
- General analgesia: Paracetamol, Paracetamol/orphenadrine
- Non-steroidal anti-inflammatory drugs (NSAIDs): Naproxen
- Opioids: Tramadol, Tramadol/Paracetamol
- Steroids: Prednisolone
Preventive medication
- Tricyclic antidepressants (TCAs): Amitriptyline, Nortriptyline
- Serotonin and norepinephrine reuptake inhibitors (SNRIs): Venlafaxine
- Angiotensin receptor blockers (ARBs): Candesartan
- Beta-blockers: Propranolol, Atenolol, Metoprolol
- Anti-epileptics: Topiramate, Valproate
- Calcium channel blockers (CCBs): Flunarizine
**Specific migraine**
Acute medication
- Ergots: Ergotamine derivatives
- Triptans: Sumatriptan, Eletriptan, Zolmitriptan
Preventive medication
- Calcitonin gene-related peptide (CGRP) monoclonal antibody (mAbs): Erenumab, Galcanezumab

Medications used for identifying each variable from the EHRs

**Medications used in ER for acute migraine headache attack**
- Ketorolac inj.
- Parecoxib inj.
- Dexamethazone inj.
- Tramadol inj.
- Morphine inj.
- Pethidine inj.
- Diclofenac inj.
**Medications used in depression/anxiety**
Tricyclic antidepressants (TCAs)
- Amitriptyline
- Imipramine
- Maprotiline
- Nortriptyline
Selective serotonin reuptake inhibitors (SSRIs)
- Escitalopram
- Paroxetine
- Sertraline
- Fluoxetine
- Fluvoxamine
Serotonin and norepinephrine reuptake inhibitors (SNRIs)
- Desvenlafaxine
- Duloxetine
- Venlafaxine
Noradrenergic and specific serotonergic antidepressants (NASSAs)
- Mianserin
- Mirtazapine
Norepinephrine and dopamine reuptake inhibitors (NDRIs)
- Bupropion

- Buspirone<br>Serotonin antagonist and reuptake inhibitors (SARIs)<br>- Trazodone<br>Norepinephrine reuptake inhibitors (NRIs)<br>- Reboxetine<br>Serotonin and dopamine antagonists (SDAs)<br>- Quetiapine<br>Multimodal antidepressants<br>- Vortioxetine<br>Monoamine oxidase inhibitors (MAOIs)<br>- Moclobemide<br>Melanogenic agonists<br>- Agomelatine<br>- Melatonin<br>Benzodiazepines (BZDs)<br>- Alprazolam<br>- Bromazepam<br>- Clonazepam<br>- Chlordiazepoxide<br>- Diazepam<br>- Clorazepate<br>- Lorazepam<br>- Midazolam<br>- Triazolam<br>Non-Benzodiazepines (Non-BZDs)<br>- Zolpidem
**Medications used in constipation**
Stimulant laxatives<br>- Bisacodyl<br>- Sennosides<br>Osmotic laxatives<br>- Lactulose<br>- Magnesium hydroxide<br>Bulk laxatives<br>- Psyllium<br>Ileal bile acid transporter inhibitor<br>- Elobixibat<br>Chloride channel activator<br>- Lubiprostone<br>Serotonin (5-HT4) receptor agonists<br>- Prucalopride
**Combined oral contraceptives (COCs)**
- Desogestrel/Ethinyl estradiol<br>- Estradiol/Nomegestrol Acetate<br>- Ethinyl estradiol/Chlormadinone acetate<br>- Ethinyl estradiol/Cyproterone acetate<br>- Ethinyl estradiol/Norgestimate<br>- Ethinyl estradiol/Desogestrel<br>- Ethinyl estradiol/Drospirenone<br>- Ethinyl estradiol/Gestodene<br>- Ethinyl estradiol/Levonorgestrel
**Preventive migraine medications**
Non-anti-seizure medication (Non-ASM)<br>- Propranolol<br>- Metoprolol

- Atenolol
- Amitriptyline
- Nortriptyline
- Flunarizine
- Venlafaxine
- Candesartan
Anti-seizure medication (ASM)
- Topiramate
- Valproate
Calcitonin gene-related peptide (CGRP) monoclonal antibody (mAbs)
- Erenumab
- Galcanezumab

Table S2 Description of data sources for constructing the cohort in the study

Data source	Description
**EHR feature domains**	
Demographic	The table retains demographic information of patients, such as gender, nationality, marital status, DOB.
Visit	The table retains records of all patient encounters with OPD, short-stay units, and ER. It contains relevant information for each visit, such as date and time, insurance, clinic, and type of service.
Admission	The table retains records of all patient encounters with IPD. It contains relevant information for each admission, such as date and time, insurance, ward of admission, and length of stay.
Vital sign	The table retains records of vital signs measured in patients across all encounter types. It contains relevant information, such as BMI, blood pressure, heart rate, body temperature.
Diagnosis	The table retains records of all diseases diagnosed in patients across all encounter types. Diseases are coded following ICD-10 and also contains other related information.
Procedure	The table retains records of all medical procedures undergone by patients across all encounter types. Procedures are coded following ICD-9-CM and also contains other related information.
Medication	The table retains records of all medications prescribed to patients across all encounter types. Medications are coded using local drug codes, which are linked to extended tables associated with other coding systems, including TMT, ATC, and MIMS. It also includes relevant information such as dose, dosage unit, frequency, and route of administration.
Bill	The table retains records of all payment information for patients across all encounter types. The service codes are coded using local codes, which may include procedure codes and drug codes. It also includes relevant information such as item/service codes, expense, discount, insurance.
**Other sources**	
CEB Data warehouses	CEB Data warehouses contain longitudinal cohorts utilizing RWD for NCDs, including HT, CKD, DM. HT patients were identified through rigorous steps involving ICD-10 and medications. The cohort was constructed from EHR data sourced from HIS and is organized for analysis under CEB*. The Data warehouses also contain patients diagnosed with OSA from CPAP/PSG reports and OSA patients who treated with CPAP.
HC	HC is responsible for managing all aspects of human resources. Data from HC includes information related to physicians, such as organizational unit, field of study, position, job, date of graduation, and date of employment at the hospital.

*Department of Clinical Epidemiology and Biostatistics (CEB), Faculty of Medicine, Ramathibodi Hospital, Mahidol University, Thailand.

Abbreviations: ATC, Anatomical Therapeutic Chemical; BMI, body mass index; CKD, chronic kidney disease; CPAP, continuous positive airway pressure; DM, diabetes mellitus; DOB, date of birth; EHR, electronic health record; ER, emergency room; HC, human capital; HIS, hospital information system; HT, hypertension; ICD-10, the International Classification of Diseases, 10th Revision codes; ICD-9-CM, the International Classification of Diseases, Ninth Revision, Clinical Modification codes; IPD, inpatient department; MIMS, Monthly Index of Medical Specialties; NCDs, non-communicable diseases; OPD, outpatient department; OSA, obstructive sleep apnea; PSG, polysomnography; RWD, real-world data; TMT, Thai Medicines Terminology

Table S3 The metadata for the variables included in the study

Variable	Data source	Original form	Definition/Measurement method
**Patient domain**			
Demographics			
Age, year	Demographic, Visit	Scale	Calculated from DOB
Gender	Demographic	Category	Sex
Marital status	Demographic	Category	Marital status
Occupation	Demographic	Category	Occupation
Education	Demographic	Category	Education
Health insurance	Visit, Admission, Bill	Category	Health insurance
BMI, kg/m^2	Vital sign	Scale	BMI or calculated from body weight and height
Migraine and related characteristics			
Migraine diagnosis	Diagnosis	Category	ICD-10: G43
Duration of migraine, year	Visit, Diagnosis, Medication, Bill	Scale	Calculated from the date of first migraine diagnosis to the current visit date
Concomitant of TTH/CH	Diagnosis	Category	ICD-10: G44.0, G44.2
Headache attacks visiting ER	Visit, Medication, Bill	Category	Visited ER department AND received medications related to acute migraine attack treatments*
History of MO/MOH	Diagnosis, Medication, Bill	Category	Detailed in Table S5
Comorbidities and symptoms			
Depression/anxiety	Diagnosis, Medication, Bill	Category	ICD-10: F32 AND medications related to depression/anxiety treatments*
Traumatic head injury	Diagnosis, Procedure	Category	Included<br>ICD-10: S02.0, S02.1, S02.8, S02.9, S04.2, S04.3, S04.4, S06.0, S06.1, S06.2, S06.3, S06.4, S06.5, S06.6, S06.7, S06.8, S06.9, S07.1<br>ICD-9-CM: 0110, 012, 0131, 0139, 0151, 0159, 0202, 0211, 0212<br>Excluded<br>ICD-10: C71, G06.0, G35, G93.6, I60, I61, I62, I67.1
OSA	Diagnosis, CEB Data warehouses	Category	ICD-10: G47.3 OR CPAP/PSG reports
HT	CEB Data warehouses	Category	ICD-10: I10, I11, I12, I13, I15, I11.0, I11.9, I12.0, I12.9, I13.0, I13.1, I13.2, I13.9, I15.0, I15.1, I15.2, I15.8, I15.9 AND medications related to HT treatments
CVD	Diagnosis, Procedure	Category	ICD-10: I20, I21, I22, I23, I24.0, I24.8, I24.9, I25.0, I25.1, I25.2, I25.5, I25.6, I25.8, I25.9, I50.0, I50.1, I50.9, I51.6, I51.7, I60, I61, I62, I63, I65.0, I65.1, I65.2, I67.0, I67.1, I67.2, I67.3, I67.4, I67.8, I67.9, I68.8, I69.0, I69.1, I69.2, I69.3, I69.4, I69.8, G46<br>ICD-9-CM: 0040, 0041, 0042, 0043, 0044, 0045, 0046, 0047, 0048, 3600, 3601, 3602, 3603, 3604, 3605, 3610, 3611, 3612, 3613, 3614,

			3615, 3616, 3617, 3619, 3732, 3810, 3811, 3812, 3813, 3814, 3815, 3816, 3818
Constipation	Diagnosis, Medication, Bill	Category	ICD-10: K59.0 OR medications related to constipation treatments*
Medication			
COCs	Medication, Bill	Category	Medications related to COCs*
**Physician domain**			
Position	Diagnosis, Medication, Bill, HC	Category	Position and job
Graduation period	Diagnosis, Medication, Bill, HC	Category	The date of graduation or first employment at the hospital, categorized as either before or after 2006
Year of experience, year	Visit, Diagnosis, Medication, Bill, HC	Scale	Calculated from the date of graduation or first employment at the hospital to the current visit date
Clinic type	Visit	Category	Hospital department visited by the patient
**Treatment domain**			
Received preventive migraine medications	Medication, Bill	Category	Medications related to preventive migraine treatments*
Time to initially start preventive migraine medications, month	Visit, Diagnosis, Medication, Bill	Scale	Calculated from the date of the first migraine diagnosis to the date of initial start of preventive migraine medications*
Type of preventive migraine medications	Medication, Bill	Category	Medications related to preventive migraine treatments*

*See Table S1 for the full list of medications

Abbreviations: BMI, body mass index; CEB, Department of Clinical Epidemiology and Biostatistics; CH, cluster headache; COCs, combined oral contraceptives; CPAP, continuous positive airway pressure; CVD, cardiovascular diseases; DOB, date of birth; ER, emergency room; HC, human capital; HT, hypertension; ICD-10, the International Classification of Diseases, 10th Revision codes; ICD-9-CM, the International Classification of Diseases, Ninth Revision, Clinical Modification codes; kg, kilogram; m, meter; MO, medication overuse; MOH, medication overuse; OSA, obstructive sleep apnea; PSG, polysomnography; TTH, tension-type headache

Table S4 Structure of the data retrieved from the EHRs

<u>Demographic domain</u>

Column header	Description	Note
ENC_HN	Hashed HN	
H2L1KEY	Sex	M=male, F=female
H4L1KEY	Nationality	*Convert text in "H4L1KEY" and "Mnemonic" to uppercase before mapping<br>Mapping table: Country<br>Key: Mnemonic
H5L1KEY	Ethnicity	*Convert text in "H4L1KEY" and "Mnemonic" to uppercase before mapping<br>Mapping table: Country<br>Key: Mnemonic
H6L1KEY	Province code	Internal code
H6L1DES	Province	
H7L1DES	Occupation	
H8L1KEY	Marital status code	
H8L1DES	Marital status	
H9L1KEY	Residence code	
H9L1DES	Residence (Thai, Non-Thai)	
D020AT3	Date of birth	Data format: "YYYYMMDD"
DISTRICT_CODE	District code	*Internal code<br>Mapping table: DistrictRama<br>Key: Mnemonic
ZIPCODE	Zipcode	

<u>Visit domain</u>

Column header	Description	Note
ENC_HN	Hashed HN	
D001KEY	Date of visit	Data format: "YYYYMMDD"
D001IN	Timestamp of patient in room	Data format: "YYYYMMDD hh:mm:ss"
D001OUT	Timestamp of patient out room	Data format: "YYYYMMDD hh:mm:ss"
D021KEY	Encounter type	Mapping table: D021<br>Key: D021KEY
D022KEY	Health insurance	Mapping table: D022<br>Key: D022KEY
D032KEY	Type of service	Mapping table: D032<br>Key: D032KEY
D108KEY	Clinic	Mapping table: D108<br>Key: D108KEY
REMARK1	Encounter ID	

<u>Admission domain</u>

Column header	Description	Note
ENC_HN	Hashed HN	
D001KEY	Date of discharge from hospital	Data format: "YYYYMMDD"
D022KEY	Health insurance	Mapping table: D022<br>Key: D022KEY
D023KEY	Type of discharge	Mapping table: D023<br>Key: D023KEY

D037KEY	DRG code	Mapping table: D037<br>Key: D037KEY
D108KEY	Ward of discharge	Mapping table: D108<br>Key: D108KEY
M1048	Length of stay	Number of days between date of admission and date of discharge
M1052	Relative weight (RW)	
M1053	Adjusted relative weight (Adjusted RW)	
REMARK1	Admission number (AN)	
ATIME	Time of admission	Data format: "hh:mm"
ADATE	Date of admission	Data format: "YYYYMMDD"
DTIME	Time of discharge from hospital	Data format: "hh:mm"
AWARD	Ward of admission	Mapping table: D108<br>Key: D108KEY

Vital sign domain

Column header	Description	Note
ENC_HN	Hashed HN	
RECORDDATE	Date of recording	Data format: "YYYYMMDD"
RECORDTIME	Time of recording	Data format: "h:m, tt"
AVPU	Vital sign test	See description at table "F0_VITALSIGN_PHYSICAL_MASTER"
REWS	Vital sign test	See description at table "F0_VITALSIGN_PHYSICAL_MASTER"
ACT	Vital sign test	See description at table "F0_VITALSIGN_PHYSICAL_MASTER"
HR	Vital sign test	See description at table "F0_VITALSIGN_PHYSICAL_MASTER"
TEMP	Vital sign test	See description at table "F0_VITALSIGN_PHYSICAL_MASTER"
SPO2	Vital sign test	See description at table "F0_VITALSIGN_PHYSICAL_MASTER"
BMI	Vital sign test	See description at table "F0_VITALSIGN_PHYSICAL_MASTER"
SBP	Vital sign test	See description at table "F0_VITALSIGN_PHYSICAL_MASTER"
HIGH	Vital sign test	See description at table "F0_VITALSIGN_PHYSICAL_MASTER"
MAP	Vital sign test	See description at table "F0_VITALSIGN_PHYSICAL_MASTER"
DBP	Vital sign test	See description at table "F0_VITALSIGN_PHYSICAL_MASTER"
RESP	Vital sign test	See description at table "F0_VITALSIGN_PHYSICAL_MASTER"
BW	Vital sign test	See description at table "F0_VITALSIGN_PHYSICAL_MASTER"

Diagnosis domain

Column header	Description	Note
ENC_HN	Hashed HN	
D001KEY	Date of diagnosis	Data format: "YYYYMMDD"

D021KEY	Encounter type	Mapping table: D021<br>Key: D021KEY
D035KEY	Diagnosis (ICD-10)	Mapping table: D035<br>Key: D035KEY
D108KEY	Clinic/Ward	Mapping table: D108<br>Key: D108KEY
D195KEY	Sequence number of ICD-10	1=primary diagnosis<br>other number = secondary diagnosis
REMARK1	Encounter ID	Equal to admission number (AN) if D021KEY="IMP"

Procedure domain

Column header	Description	Note
ENC_HN	Hashed HN	
D001KEY	Date of diagnosis	Data format: "YYYYMMDD"
D021KEY	Encounter type	Mapping table: D021<br>Key: D021KEY
D036KEY	Operation code (ICD-9-CM)	Mapping table: D036<br>Key: D036KEY
D108KEY	Clinic/Ward	Mapping table: D108<br>Key: D108KEY
D195KEY	Sequence number of ICD-9-CM	1=primary operation<br>other number = secondary operation
REMARK1	Encounter ID	Equal to admission number (AN) if D021KEY="IMP"

Medication domain

Column header	Description	Note
ENC_HN	Hashed HN	
RQSeq	Sequence number of drug dispensing	
DSPCode	Drug code	Mapping table: D403<br>Key: D403KEY
UOM	Unit of dose	
NED	Reason for prescribing non-essential drugs (NED)	Mapping table: D411<br>Key: D411KEY
ISSQTY	Quantity dispensed	
PerformDate	Timestamp of dispensing	Data format: "YYYY-MM-DD hh:mm, tt"
REMAINQTY	Remain quantity	
ENCID	Encounter ID	Equal to admission number (AN) if D021KEY="IMP"
ENCType	Encounter type	Mapping table: D021<br>Key: D021KEY
OrderUnit	Order unit	Mapping table: D108<br>Key: D108KEY
ClinicType	Type of service	Mapping table: D032<br>Key: D032KEY
DoseSeq	Sequence number of dose	
Dose	Number of dose	
DoseUnit	Unit of dose	
Frequency	Dose frequency	
Method	before/after meal	
Route	Drug route	

Site	Drug site	
PRN		
AdminDay	Day of taking drug	
AdminTime	Time of taking drug	
Duration	Dose duration	
Comment	Detail of drug regimen	Free text

<u>Bill domain</u>

**Column header**	**Description**	**Note**
ENC_HN	Hashed HN	
D001KEY	Billing date	Data format: "YYYYMMDD"
D021KEY	Encounter type	Mapping table: D021<br>Key: D021KEY
D022KEY	Health insurance (for each item)	Mapping table: D022<br>Key: D022KEY
D032KEY	Type of service	Mapping table: D032<br>Key: D032KEY
D033KEY	Item/service code	Mapping table: D033<br>Mapping condition should follow 1), otherwise it will follow 2)<br>1) D033KEY=D033KEY and D001KEY between START_DATE and END_DATE<br>2) D033KEY=D033KEY with the record of latest END_DATE
D039KEY	Discount code	Mapping table: D039<br>Key: D039KEY
D108KEY	Request unit	Mapping table: D108<br>Key: D108KEY
D108KEY_1	Perform unit	Mapping table: D108<br>Key: D108KEY
M1022	Quantity of item or times of service	Data format: decimal number
M1030	Expense	Data format: decimal number
M1045	Discount	Data format: decimal number
REMARK1	Encounter ID	Equal to admission number (AN) if D021KEY="IMP"

Table S5 Definitions and measurement methods for outcome in the study

Outcome	Data source	Original form	Definition/Measurement method
MO/MOH	Diagnosis, Medication, Bill	Category	**ICD-10**<br>- G44.4 (Drug-induced headache, not elsewhere classified)<br>**Medication**<br>Non-specific migraine medications<br>- Paracetamol<br>- NSAIDs: diclofenac, sulindac, diflunisal, parecoxib, indomethacin, nabumetone, piroxicam, ibuprofen, ketorolac, mefenamic acid, meloxicam, naproxen, tenoxicam, etoricoxib, celecoxib<br>Specific migraine medications<br>- Opioids: tramadol, tramadol/paracetamol, morphine, fentanyl, methadone, pethidine<br>- Ergots: ergotamine derivatives<br>- Triptans: sumatriptan, eletriptan, zolmitriptan

Abbreviations: ICD-10, the International Classification of Diseases, 10th Revision codes; MO, medication overuse; MOH, medication overuse headache; NSAIDs, non-steroidal anti-inflammatory drugs

Table S6 Missing assessment of variables and characteristics of patients at index assessment for those included in the final analysis and those excluded due to missing variable data

Variable	Count of non-missing data	Percentage of non-missing data	Complete cases	Excluded cases due to missing data
**No. of patients**	14,251	100.0	14,227	24
**Patient domain**				
Demographics				
Age, year, mean (SD)	14,251	100.0	45.4 (14.1)	45.3 (14.2)
Male, n (%)	14,251	100.0	2131 (15.0)	3 (14.4)
Marital status, n (%)	14,251	100.0		
Married			6900 (48.5)	11 (45.8)
Unmarried			6026 (42.4)	11 (45.8)
Separated/Divorced/ Widowed			1301 (9.1)	2 (8.3)
Occupation, n (%)	14,241	99.9		
Worker			10561 (74.2)	
Student			1357 (9.5)	
Housewife			2142 (15.1)	
Retirement			167 (1.2)	
Education, n (%)	14,241	99.9		
Primary school			1868 (13.1)	
High school			4468 (31.4)	
Bachelor/Higher degree			7891 (55.5)	
Health insurance, n (%)	14,251	100.0		
UHC/Social securities			3920 (27.6)	7 (29.2)
Civil servant/Direct contact			6346 (44.6)	10 (41.7)
Self-payment			3961 (27.8)	7 (29.2)
BMI, kg/m², n (%)	14,227	95.4		
≤18.4			1066 (7.5)	
18.5-24.9			7816 (54.9)	
25.0-29.9			3746 (26.3)	
≥30			1599 (11.2)	
Migraine and related characteristics				
Migraine diagnosis, n (%)	14,251	100.0		
Migraine with aura			188 (1.3)	0 (0.0)
Migraine without aura			386 (2.7)	1 (4.2)
Unspecified migraine			13653 (96.0)	23 (95.8)
Duration of migraine, year, n (%)	14,251	100.0		
<1			12394 (87.1)	21 (87.5)
1-5			1368 (9.6)	2 (8.3)
≥6			465 (3.3)	1 (4.2)
Concomitant of TTH/CH, n (%)	14,251	100.0	462 (3.2)	1 (4.2)
Headache attacks visiting ER, n (%)	14,251	100.0	449 (3.2)	2 (8.3)
History of MO/MOH, n (%)	14,251	100.0	873 (6.1)	2 (8.3)
Comorbidities and symptoms				
Depression/Anxiety, n (%)	14,251	100.0	6638 (46.7)	11 (45.8)
Traumatic head injury, n (%)	14,251	100.0	77 (0.5)	0 (0.0)
OSA, n (%)	14,251	100.0	384 (2.7)	1 (4.2)
HT, n (%)	14,251	100.0	4342 (30.5)	8 (33.3)
CVD, n (%)	14,251	100.0	787 (5.5)	2 (8.3)
Constipation, n (%)	14,251	100.0	296 (2.1)	1 (4.2)
Medication				
COCs, n (%)	14,251	100.0	824 (5.8)	2 (8.3)

**Physician domain**				
Position, n (%)	14,251	100.0		
Hospitalist/Intern			1915 (13.5)	3 (12.5)
Resident/Fellow			7213 (50.7)	12 (50.0)
Staff			5099 (35.8)	9 (37.5)
Graduation period, n (%)	14,251	100.0		
Before 2006			5829 (41.0)	10 (41.7)
After 2006			8398 (59.0)	14 (58.3)
Year of experience, year, median (IQR)	14,251	100.0	7.0 (1.0-15.0)	7.0 (1.0-15.0)
Clinic type, n (%)	14,251	100.0		
Neurology specialist clinic			1143 (8.0)	2 (8.3)
Family medicine clinic			5669 (39.8)	9 (37.5)
Non-neurology internal medicine specialist clinic			2781 (19.5)	5 (20.8)
Other specialist clinic			4634 (32.6)	8 (33.3)
**Treatment domain**				
Preventive migraine medications, n (%)				
No			6767 (47.6)	12 (50.0)
Yes			7460 (52.4)	12 (50.0)
Time to initially start preventive migraine medications, month, n (%)	14,251	100.0		
≤6			5140 (36.1)	8 (33.3)
7-12			487 (3.4)	1 (4.2)
>12			1833 (12.9)	3 (12.5)
Type of preventive migraine medications, n (%)	14,251	100.0		
Non-ASM			6219 (43.7)	10 (41.7)
ASM			750 (5.3)	1 (4.2)
CGRP mAbs/ Combinations			491 (3.5)	1 (4.2)

Abbreviations: ASM, anti-seizure medication; BMI, body mass index; CVD, cardiovascular diseases; CGRP, calcitonin gene-related peptide; CH, cluster headache; COCs, combined oral contraceptives; ER, emergency room; HT, hypertension; IQR, interquartile range; kg, kilogram; m, meter; mAbs, monoclonal antibodies; MO, medication overuse; MOH, medication overuse headache; n, number; OSA, obstructive sleep apnea; SD, standard deviation; TTH, tension-type headache; UHC, universal health coverage

Table S7 Variable type and transformation

Variable	Data type	Original form		Model required form	
		Form	Value	Form	Value
**Patient domain**					
Demographics					
Age, year	T	Scale	≥18	Scale	≥18
Gender	C	Category	Male, Female	Category	0, 1
Marital status	C	Category	Married, Unmarried, Separated/Divorced/Widowed	Category	One-hot-encoding
Occupation	C	Category	Worker, Student, Housewife, Retirement	Category	One-hot-encoding
Education	C	Category	Primary school, High school, Bachelor/Higher degree	Category	One-hot-encoding
Health insurance	C	Category	UHC/Social Securities, Civil servant/Direct contact, Self-payment	Category	One-hot-encoding
BMI, kg/m²	T	Category	≤18.4, 18.5-24.9, 25.0-29.9, ≥30	Category	One-hot-encoding
Migraine and related characteristics					
Migraine diagnosis	T	Category	Migraine with aura, Migraine without aura, Unspecified migraine	Category	One-hot-encoding
Duration of migraine, year	T	Category	<1, 1-5, ≥6	Category	One-hot-encoding
Concomitant of TTH/CH	T	Category	No, Yes	Category	0, 1
Headache attacks visiting ER	T	Category	No, Yes	Category	0, 1
History of MO/MOH	T	Category	No, Yes	Category	0, 1
Comorbidities and symptoms					
Depression/Anxiety	T	Category	No, Yes	Category	0, 1
Traumatic head injury	T	Category	No, Yes	Category	0, 1
OSA	T	Category	No, Yes (without CPAP), Yes (with CPAP)	Category	One-hot-encoding
HT	T	Category	No, Yes	Category	0, 1
CVD	T	Category	No, Yes	Category	0, 1
Constipation	T	Category	No, Yes	Category	0, 1
Medication					
COCs	T	Category	No, Yes	Category	0, 1
**Physician domain**					
Position	T	Category	Hospitalist/Intern, Resident/Fellow, Staff	Category	One-hot-encoding
Graduation period	T	Category	Before 2006, After 2006	Category	0, 1
Year of experience, year	T	Scale	≥1	Scale	≥1
Clinic type	T	Category	Neurology specialist clinic, Family medicine clinic, Non-neurology internal	Category	One-hot-encoding

			medicine specialist clinic, Other specialist clinic		
**Treatment domain**					
Time to initially start preventive migraine medications, month	T	Category	Not received, ≤6 months, 7-12 months, >12 months	Category	One-hot-encoding
Type of preventive migraine medications	T	Category	Not received, Non-ASM, ASM, Combinations/CGRP mAbs	Category	One-hot-encoding

Abbreviations: ASM, anti-seizure medication; BMI, body mass index; C, constant variable; CGRP, calcitonin gene-related peptide; CH, cluster headache; COCs, combined oral contraceptives; CPAP, continuous positive airway pressure; CVD, cardiovascular diseases; ER, emergency room; HT, hypertension; kg, kilogram; m, meter; mAbs, monoclonal antibodies; MO, medication overuse; MOH, medication overuse headache; OSA, obstructive sleep apnea; T, time-varying variable; TTH, tension-type headache; UHC, universal health coverage

Table S8 Lists of hyperparameters for tuning the RSF and XGBoost models

Hyperparameter	Value*
**Full model**	
RSF model	
Maximum depth of each tree (max_depth)	4, 8, 10, **12**
Number of trees in the forest (n_estimators)	**32**, 64, 128, 256, 512, 1024
Number of leaf nodes in the tree (max_leaf_nodes)	10, 20, 30, **40**, 50, 60, 70, 80, 90, 100
Minimum number of samples required to split an internal node (min_samples_split)	5, 10, 15, 20, **25**, 30
Number of features to consider when splitting a node (max_features)	3, 6, 9, 12, 15, **18**, "sqrt"
XGBoost model	
Learning rate (learning_rate)	**0.2**, 0.1, 0.05, 0.01
Maximum depth of a tree (max_depth)	4, 6, 8, 10, **12**
**Reduced model**	
RSF model	
Maximum depth of each tree (max_depth)	4, **8**, 10, 12
Number of trees in the forest (n_estimators)	**32**, 64, 128, 256, 512, 1024
Number of leaf nodes in the tree (max_leaf_nodes)	10, 20, 30, **40**, 50, 60, 70, 80, 90, 100
Minimum number of samples required to split an internal node (min_samples_split)	5, **10**, 15, 20, 25, 30
Number of features to consider when splitting a node (max_features)	3, 6, 9, **12**, 15, 18, "sqrt"
XGBoost model	
Learning rate (learning_rate)	**0.2**, 0.1, 0.05, 0.01
Maximum depth of a tree (max_depth)	4, 6, 8, 10, **12**

*The best hyperparameters are bolded.
Abbreviations: RSF, random survival forests; XGBoost, extreme gradient boosting

Table S9 Number of patients corresponding to each number of MO/MOH events experienced

Event of MO/MOH	Number of patients	Percentage
1	2,224	56.58
2	825	20.99
3	391	9.95
4	205	5.21
5	120	3.05
6	69	1.76
7	37	0.94
8	33	0.84
9	16	0.41
10	3	0.08
11	5	0.13
12	1	0.03
13	0	0.00
14	1	0.03
15	1	0.03

Abbreviations: MO, medication overuse; MOH, medication overuse headache

Table S10 Univariate CPH model on training dataset

Variable	MO/MOH (n = 2754)	Non-MO/MOH (n = 7711)	HR (95% CI)	P-value
**Percentage**	26.3	73.7		
**Patient domain**				
Demographics				
Age, year, mean (SD)	46.4 (14.1)	45.1 (15.2)	1.00 (0.99-1.00)	0.080
Gender, n (%)				0.059
Male	367 (13.3)	1184 (15.4)	1	
Female	2387 (86.7)	6527 (84.6)	1.10 (1.00-1.21)	
Marital status, n (%)				0.002
Married	1461 (53.1)	3638 (47.2)	1	
Unmarried	1012 (36.7)	3381 (43.8)	0.87 (0.81-0.94)	
Separated/Divorced/Widowed	281 (10.2)	692 (9.0)	1.03 (0.92-1.15)	
Occupation, n (%)				0.001
Worker	2095 (76.1)	5671 (73.5)	1	
Student	191 (6.9)	813 (10.5)	0.79 (0.70-0.90)	
Housewife	436 (15.8)	1129 (14.6)	1.05 (0.96-1.16)	
Retirement	32 (1.2)	98 (1.3)	0.92 (0.67-1.27)	
Education, n (%)				0.080
Primary school	408 (14.8)	1001 (13.0)	1	
High school	867 (31.5)	2440 (31.6)	0.89 (0.80-0.99)	
Bachelor/Higher degree	1479 (53.7)	4270 (55.4)	0.90 (0.81-0.99)	
Health insurance, n (%)				<0.001
UHC/Social securities	701 (25.5)	2215 (28.7)	1	
Civil servant/Direct contact	1430 (51.9)	3251 (42.2)	1.29 (1.19-1.40)	
Self-payment	623 (22.6)	2245 (29.1)	1.05 (0.95-1.16)	
BMI, kg/m², n (%)				<0.001
≤18.4	123 (4.5)	652 (8.5)	1	
18.5-24.9	1434 (52.1)	4301 (55.8)	1.49 (1.27-1.74)	
25.0-29.9	801 (29.1)	1959 (25.4)	1.64 (1.39-1.94)	
≥30	396 (14.4)	799 (10.4)	1.91 (1.59-2.28)	
Migraine and related characteristics				
Migraine diagnosis, n (%)				0.700
Migraine with aura	32 (1.2)	97 (1.3)	1	
Migraine without aura	68 (2.5)	206 (2.7)	0.93 (0.65-1.33)	
Unspecified migraine	2654 (96.4)	7408 (96.1)	1.02 (0.75-1.38)	
Duration of migraine, year, n (%)				<0.001
<1	2389 (86.7)	6754 (87.6)	1	
1-5	315 (11.4)	669 (8.7)	1.13 (1.03-1.25)	
≥6	50 (1.8)	288 (3.7)	0.70 (0.56-0.89)	
Concomitant of TTH/CH, n (%)				0.063
No	2678 (97.2)	7430 (96.4)	1	
Yes	76 (2.8)	281 (3.6)	0.82 (0.67-1.01)	
Headache attacks visiting ER, n (%)				0.203
No	2653 (96.3)	7494 (97.2)	1	
Yes	101 (3.7)	217 (2.8)	1.12 (0.94-1.32)	
History of MO/MOH, n (%)				<0.001
No	2447 (88.9)	7374 (95.6)	1	
Yes	307 (11.1)	337 (4.4)	2.16 (1.97-2.38)	
Comorbidities and symptoms				
Depression/Anxiety, n (%)				<0.001
No	1286 (46.7)	4418 (57.3)	1	
Yes	1468 (53.3)	3293 (42.7)	1.49 (1.40-1.58)	
Traumatic head injury, n (%)				0.030
No	2744 (99.6)	7661 (99.4)	1	
Yes	10 (0.4)	50 (0.6)	0.56 (0.31-1.03)	
Obstructive sleep apnea, n (%)				0.007
No	2696 (97.9)	7489 (97.1)	1	

Yes (without CPAP)	55 (2.0)	192 (2.5)	0.81 (0.64-1.03)	
Yes (with CPAP)	3 (0.1)	30 (0.4)	0.30 (0.07-1.32)	
Hypertension, n (%)				<0.001
No	1734 (63.0)	5548 (71.9)	1	
Yes	1020 (37.0)	2163 (28.1)	1.37 (1.28-1.47)	
Cardiovascular diseases, n (%)				0.353
No	2595 (94.2)	7290 (94.5)	1	
Yes	159 (5.8)	421 (5.5)	0.93 (0.80-1.08)	
Constipation, n (%)				0.119
No	2695 (97.9)	7562 (98.1)	1	
Yes	59 (2.1)	149 (1.9)	1.19 (0.96-1.49)	
Medication				
COCs, n (%)				0.019
No	2625 (95.3)	7235 (93.8)	1	
Yes	129 (4.7)	476 (6.2)	0.83 (0.71-0.97)	
**Physician domain**				
Position, n (%)				<0.001
Hospitalist/Intern	382 (13.9)	1011 (13.1)	1	
Resident/Fellow	1277 (46.4)	4025 (52.2)	0.83 (0.76-0.94)	
Staff	1095 (39.8)	2675 (34.7)	1.12 (1.01-1.24)	
Graduation period, n (%)				0.001
Before 2006	1223 (44.4)	3068 (39.8)	1	
After 2006	1531 (55.6)	4643 (60.2)	0.89 (0.83-0.95)	
Year of experience, year, median (IQR)	7.0 (1.0-15.0)	7.0 (1.0-16.0)	1.00 (0.99-1.00)	0.830
Clinic type, n (%)				<0.001
Neurology specialist clinic	345 (12.5)	499 (6.5)	1	
Family medicine clinic	918 (33.3)	3203 (41.5)	0.47 (0.42-0.53)	
Non-neurology internal medicine specialist clinic	708 (25.7)	1339 (17.4)	0.73 (0.65-0.83)	
Other specialist clinic	783 (28.4)	2670 (34.6)	0.42 (0.38-0.47)	
**Patient-care domain**				
Received preventive migraine medications, n (%)				
No	1105 (40.1)	4108 (53.3)	1	
Time to initially start preventive migraine medications, month, n (%)				<0.001
≤6	1169 (42.4)	2429 (31.5)	1.57 (1.05-1.64)	
7-12	115 (4.2)	217 (2.8)	1.49 (1.27-1.74)	
>12	365 (13.3)	957 (12.4)	1.28 (1.18-1.40)	
Type of preventive migraine medications, n (%)				<0.001
Non-ASM	1319 (47.9)	3045 (39.5)	1.43 (1.37-1.48)	
ASM	198 (7.2)	347 (4.5)	1.67 (1.46-1.90)	
CGRP mAbs/Combinations	132 (4.8)	211 (2.7)	2.07 (1.79-2.41)	

Abbreviations: ASM, anti-seizure medication; BMI, body mass index; CGRP, calcitonin gene-related peptide; CH, cluster headache; COCs, combined oral contraceptives; CPAP, continuous positive airway pressure; ER, emergency room; HR, hazard ratio; IQR, interquartile range; kg, kilogram; m, meter; mAbs, monoclonal antibodies; MO, medication overuse; MOH, medication overuse headache; n, number; SD, standard deviation; TTH, tension-type headache; UHC, universal health coverage

Table S11 Variable importance of CPH model using likelihood ratio test on training dataset

Full model

Predictive variables	LRT	Df	P-value
Clinic type	149.80	3	<0.000001
History of MO/MOH	108.78	1	<0.000001
Physician position	69.95	2	<0.000001
Insurance	40.02	2	<0.000001
BMI, kg/m^2	37.86	3	<0.000001
Hypertension	28.99	1	<0.000001
Duration of migraine, year	27.47	2	0.000001
Type of preventive migraine medications	26.34	3	0.000001
Depression/Anxiety	18.12	1	0.000021
Age, year	14.48	1	0.001204
Education	12.93	2	0.001559
Concomitant of TTH/CH	6.84	1	0.015680
OSA	6.51	2	0.038492
Graduation period	4.03	1	0.044654
Gender	3.81	1	0.047966

Abbreviations: BMI, body mass index; CH, cluster headache; kg, kilogram; m, meter; MO, medication overuse; MOH, medication overuse headache; OSA, obstructive sleep apnea; TTH, tension-type headache

Reduced model

Predictive variables	LRT	Df	P-value
Clinic type	145.68	3	<0.000001
History of MO/MOH	107.99	1	<0.000001
Physician position	64.15	2	<0.000001
BMI, kg/m^2	39.64	3	<0.000001
Hypertension	29.19	1	<0.000001
Duration of migraine, year	26.65	2	<0.000001
Type of preventive migraine medications	22.54	3	0.000003
Depression/Anxiety	18.40	1	0.000018
Age, year	12.52	1	0.002420
Concomitant of TTH/CH	7.53	1	0.018666
OSA	7.02	2	0.028622
Gender	4.23	1	0.034171
Education	3.97	2	0.045110

Abbreviations: BMI, body mass index; CH, cluster headache; kg, kilogram; m, meter; MO, medication overuse; MOH, medication overuse headache; OSA, obstructive sleep apnea; TTH, tension-type headache

Table S12 CPH assumption tested with Schoenfeld residuals

Full model

Predictive variables	Chi-square	Df	P-value
History of MO/MOH	96.87	1	<0.00001
Physician position	68.04	2	<0.00001
Duration of migraine, year	48.77	1	<0.00001
Health insurance	37.88	2	<0.00001
Graduation period of physician	20.64	1	0.00001
Hypertension	11.24	1	0.00080
Clinic type	15.73	3	0.00129
Depression/Anxiety	6.57	1	0.01040
Age, year	6.20	1	0.01280
Type of preventive migraine medications	10.25	3	0.01658
OSA	5.86	2	0.05328
BMI, kg/m²	6.85	3	0.07675
Gender	1.25	1	0.26378
Concomitant of TTH/CH	0.08	1	0.77107
Education	0.46	2	0.79518
GLOBAL	246.49	27	<0.00001

Abbreviations: BMI, body mass index; CH, cluster headache; Df, degree of freedom; kg, kilogram; m, meter; MO, medication overuse; MOH, medication overuse headache; OSA, obstructive sleep apnea; TTH, tension-type headache

Reduced model

Predictive variables	Chi-square	Df	P-value
History of MO/MOH	97.55	1	<0.00001
Physician position	69.55	2	<0.00001
Duration of migraine, year	50.27	1	<0.00001
Clinic type	16.63	3	0.00084
Hypertension	10.96	1	0.00093
Depression/Anxiety	6.61	1	0.01012
Age, year	5.89	1	0.01524
Type of preventive migraine medications	10.41	3	0.01536
OSA	5.58	2	0.06129
BMI, kg/m²	6.97	3	0.07296
Gender	1.27	1	0.25973
Concomitant of TTH/CH	0.11	1	0.74017
Education	0.41	2	0.81319
GLOBAL	223.49	24	<0.00001

Abbreviations: BMI, body mass index; CH, cluster headache; Df, degree of freedom; kg, kilogram; m, meter; MO, medication overuse; MOH, medication overuse headache; OSA, obstructive sleep apnea; TTH, tension-type headache
